# Synergism of BCL-2 family inhibitors facilitates selective elimination of senescent cells

**DOI:** 10.18632/aging.204207

**Published:** 2022-08-08

**Authors:** David Rysanek, Pavla Vasicova, Jayaprakash Narayana Kolla, David Sedlak, Ladislav Andera, Jiri Bartek, Zdenek Hodny

**Affiliations:** 1 Department of Genome Integrity, Institute of Molecular Genetics of the Czech Academy of Sciences, Prague, Czech Republic; 2 CZ-OPENSCREEN, Institute of Molecular Genetics of the Czech Academy of Sciences, Prague, Czech Republic; 3 Genome Integrity Unit, Danish Cancer Society Research Center, Copenhagen, Denmark; 4 Biocev, Institute of Biotechnology of the Czech Academy of Sciences, Prague, Czech Republic

**Keywords:** homoharringtonine, cellular senescence, BCL-2, MCL-1, senolytics

## Abstract

Accumulation of senescent cells in tissues with advancing age participates in the pathogenesis of several human age-associated diseases. Specific senescent secretome, the resistance of senescent cells to apoptotic stimuli, and lack of immune system response contribute to the accumulation of senescent cells and their adverse effects in tissues. Inhibition of antiapoptotic machinery, augmented in senescent cells, by BCL-2 protein family inhibitors represents a promising approach to eliminate senescent cells from tissues. This study aimed to explore synergistic and selective senolytic effects of anti-apoptotic BCL-2 family targeting compounds, particularly BH3 mimetics.

Using human non-transformed cells RPE-1, BJ, and MRC-5 brought to ionizing radiation-, oncogene-, drug-induced and replicative senescence, we found synergy in combining MCL-1 selective inhibitors with other BH3 mimetics. In an attempt to uncover the mechanism of such synergy, we revealed that the surviving subpopulation of cells resistant to individually applied ABT-737/ABT-263, MIK665, ABT-199, and S63845 BCL-2 family inhibitors showed elevated MCL-1 compared to untreated control cells indicating the presence of a subset of cells expressing high MCL-1 levels and, therefore, resistant to BCL-2 inhibitors within the original population of senescent cells.

Overall, we found that combining BCL-2 inhibitors can be beneficial for eliminating senescent cells, thereby enabling use of lower, potentially less toxic, doses of drugs compared to monotherapy, thereby overcoming the resistance of the subpopulation of senescent cells to monotherapy.

## INTRODUCTION

Cellular senescence, a complex cellular response to stress characterized by a halt of cell cycle progression, is one factor contributing to aging and age-associated diseases. Cellular senescence can be triggered by multiple stimuli of endogenous and exogenous nature, including DNA damage provoked by oncogene activation, mitochondrial and oxidative stress, drug cytotoxicity, ionizing radiation, bacterial toxins, and viral infections [1]. There is emerging evidence that accelerated aging and age-related diseases such as chronic autoimmune inflammation, hypertension, diabetes mellitus type 2, arthritis, and cancer are negatively affected by the persistent presence of senescent cells [1]. The pathogenic effects of senescent cells are mediated by specific secreted molecules comprising peptide factors (e.g., growth factors, hormones, and cytokines; [2–4]) and reactive oxygen species [5], which subvert the tissue microenvironment. In specific tissues such as the liver, senescent cells can be cleared by the immune system [6, 7]; however, age-dependent accumulation of senescent cells was observed in other tissues, such as skin, in which the elimination of senescent cells seems to be bypassed [8, 9]. Therefore, supported by experimental models [10–12], it is believed that selective elimination of senescent cells can lead to rejuvenation of the aged organism and increase the healthspan, and as a result, clearance of senescent cells can serve as a therapeutic approach to combat many negative aspects of aging [13–15].

The age-dependent accumulation of senescent cells is caused by age-related attenuated efficiency of the immune system [16] and their higher resistance both to extrinsic and intrinsic pro-apoptotic stimuli [16–18], including oxidative stress [19]. While the mechanisms driving senescence are well studied, understanding the mechanisms endowing these cells with increased survival capacity is limited.

The BCL-2 protein family plays a central role in cell death regulation by diverse mechanisms, including apoptosis and autophagy [16, 20, 21]. This protein family, in addition to multidomain pro-apoptotic proteins Bax, Bak, and Bok and BH3-only proteins, also includes the anti-apoptotic proteins BCL-2, BCL-W, BCL-XL, MCL-1, and A1, and is intensively studied as a target for pharmacological intervention in cancer [22, 23]. Yosef et al. evaluated the contribution of individual members of the BCL-2 family and their combinations to the viability of senescent cells [24]. They found that the increased presence of BCL-W and BCL-XL underlies senescent cell resistance to apoptosis and their combined inhibition induces the death of senescent cells. This mechanism is believed to be a basis for senolytic effects of BCL-2 inhibitors such as ABT-737 or ABT-263 (Navitoclax) [24, 25].

ABT-737 and ABT-263 both display a high affinity for BCL-2, BCL-XL, and BCL-W, but not A1, or MCL-1. By binding within the hydrophobic groove of these anti-apoptotic proteins, ABT-737 and ABT-263 essentially mimic the presence of BH3-only pro-apoptotic proteins, and both compete with and displace them [26, 27]. Despite displaying excellent pro-apoptotic activity in pre-clinical studies, ABT-737 has unfavorable oral bioavailability and solubility, prompting the development of the orally available ABT-263 [27, 28]. Nevertheless, in both pre-clinical and clinical studies, Navitoclax has been found to cause thrombocytopenia, limiting its use in high doses [29, 30].

In this study, we aimed to search for synergistic selective senolytic effects using senescent RPE-1, BJ, and MRC-5 cells and their proliferating or quiescent counterparts by combining non-selective BH3 mimetics ABT-737, ABT-263 (Navitoclax) and protein synthesis inhibitory compound homoharringtonine (HHT), and selective BH3 mimetics MIK665, A1331852, ABT-199 (Venetoclax), and S63845 BCL-2 protein family inhibitors for interfering with anti-apoptotic function of BCL2, BCL-XL, and MCL-1. We found that combining selective MCL-1 inhibitors with non-MCL1 BCL-2 inhibitors results in marked synergistic effects with higher sensitivity of senescent compared to proliferating cells. These findings indicate that a combination of drugs targeting different BCL-2 family members can benefit for senolytic therapies.

## RESULTS

### Senescence models, experimental setup, and analysis

**Figure 1:**
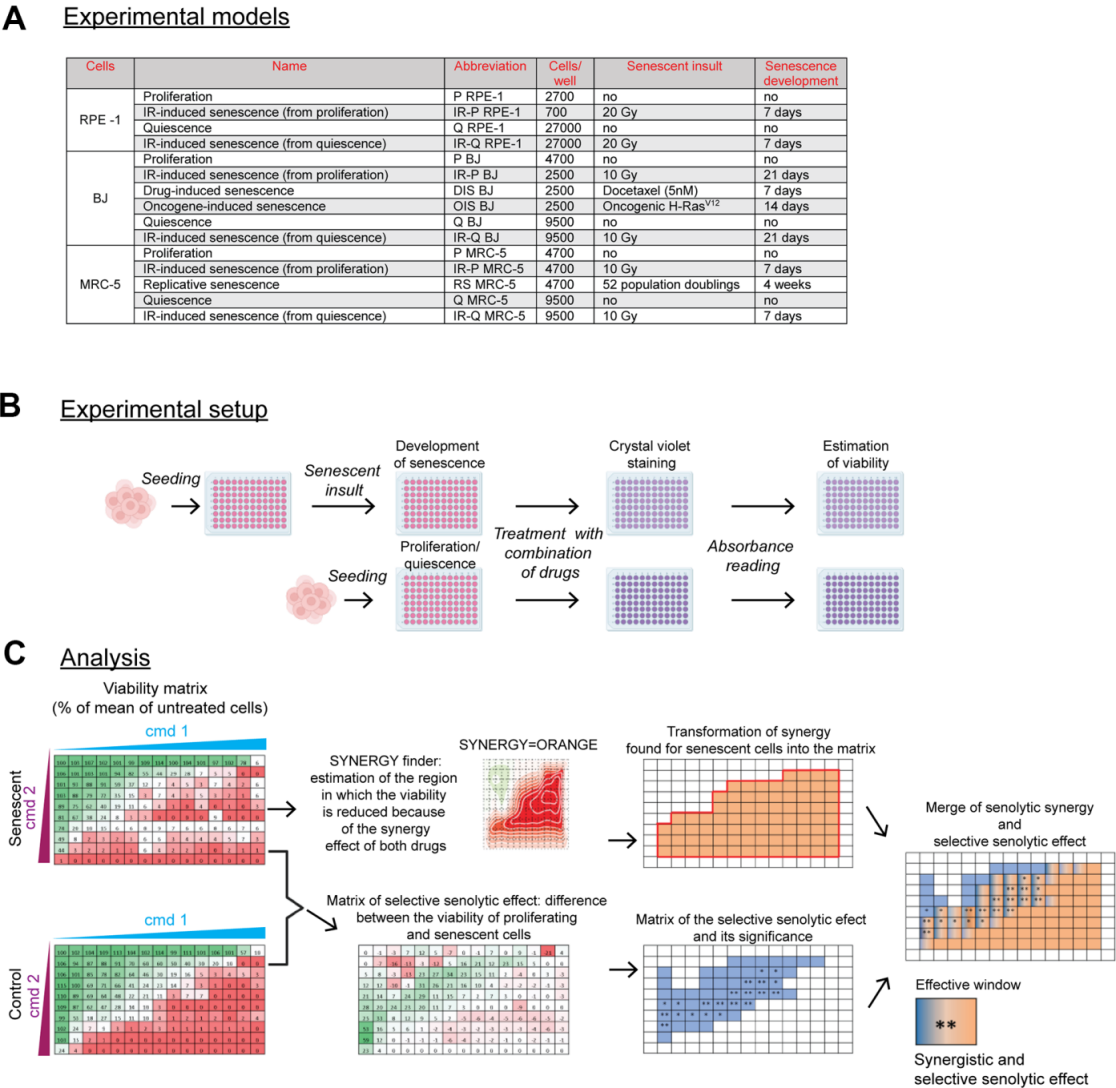
**Scheme of the experimental setup and data analysis.** (**A**) The summary of used senescence models, including their designation, the number of cells seeded, and time of senescence development. The seeding area was 0.322 cm^2^ per well of 96 well-plate. (**B**) Summary of the experimental procedure. Twenty-four hours after cell seeding, the senescent insult was applied to develop a senescent phenotype for a period depicted in (**A**). Control proliferating or quiescent cells were seeded 24 hours before the drug exposure. After 24-hour of single or combined compound treatment, the cultures were stained with crystal violet to quantify the amount of surviving cells by absorbance reading (A = 595). The cell viability was expressed as the percentage of the untreated population. (**C**) The analytical workflow. After treatment, the viability of senescent cells expressed as a percentage of a mean (n = 3) relative to untreated cells, scaled from 0 (red) to 100 percent (green), was subsequently analyzed by the SynergyFinder to determine the area of synergy for senescent cells transformed back into the matrix representing compound concentration (orange region; note that only compound combinations are presented). In parallel, the matrix of selective senolytic effect was obtained by subtracting the viability of senescent cells from the control population. The green gradient, which indicates the region where senescent cell viability was lower than the control population, was marked blue to highlight the region with a selective senolytic effect (note that only compound combinations are presented). Next, the compound concentrations giving selective senolytic effect were statistically evaluated by a two-tailed Student's t-test (*, P > 0.05; **, P < 0.01; ***, P < 0.001; ****, P < 0.0001). Finally, to obtain drug concentrations with synergistic selective senolytic effect, synergy and selective senolytic effect matrices were merged to get an effective window of compound concentrations (blue-orange fields). CMD – compound.

The main goal of this study was to find a selective senolytic effect, i.e., a scenario when the senescent cells are more sensitive than proliferating or quiescent control cells due to the synergetic effect of tested inhibitors targeting the anti-apoptotic proteins. For this purpose, we employed several well-established senescence models, including those undergoing replicative (RS), oncogene-(OIS), drug-(DIS), and ionizing radiation (IR)-induced cellular senescence. We utilized human telomerase-immortalized retinal pigment epithelial cells (RPE-1) and normal skin BJ and embryonal lung MRC-5 fibroblasts to induce senescence. We also included 'senescent' cells obtained by ionizing radiation treatment of the quiescent (Q) cell population (see Figure 1A and Material and Methods for details).

The experimental setup is depicted in Figure 1B. Briefly, the cells were seeded onto 96-well plates, and 24 hours later, the senescence-inducing insult was applied to develop a senescent phenotype (see Supplementary Figure 1 for detection of senescence-associated β-galactosidase and DNA replication activities). Next, senescent cells, with proliferating (P) and quiescent cells seeded 24 hours earlier, were exposed to combinations of tested compounds for 24 hours. The residual viability was assessed using the crystal violet staining (CV).

The analytical procedure is summarized in Figure 1C. At first, the synergy area of the tested combination was determined both for senescent and control cells using the SynergyFinder [31], and the 'area' of compound synergy for senescent cells transformed into a matrix (see the orange area, Figure 1C). Second, to determine a range of compound concentrations with selective senolytic effect, the difference in cell viability between proliferating and senescent cells was statistically evaluated for each drug concentration by the Student's t-test and expressed in the form of a matrix again (see the blue area, Figure 1C). Finally, to define a subset of compound concentrations with both the synergy and the selective senolytic effect, both matrices were merged to demarcate the effective window of compound concentrations (see the blue-orange area, Figure 1C). This experimental setup and subsequent analysis were applied to all experiments.

### Homoharringtonine augments the senolytic effects of non-selective BCL-2 inhibitors ABT-737 and ABT-263

To examine whether the combination of non-selective BCL-2 inhibitors with MCL-1 inhibitors would increase the sensitivity of senescent cells to apoptosis, we at first selected a non-specific MCL-1 inhibitor homoharringtonine (HHT) [32], which by blocking translation causes rapid cellular clearance of the short-lived MCL-1 protein [33]. HHT used alone in a concentration range of 0 – 100 nM exerted a distinct cytotoxic effect in proliferating (P RPE-1, P BJ, and P MRC-5) and quiescent (Q BJ, and Q MRC-5) cells, whereas senescent and Q RPE-1 cells appeared more resistant in general (Supplementary Figure 2A). We verified the effect of two known senolytics, ABT-263 and ABT-737, on our experimental models. As expected, senescent cells were, overall, significantly more sensitive to both compounds (Supplementary Figure 2B, 2C). The only exception was the similar sensitivity of replicative senescent and proliferating MRC-5 cells to ABT-737. Notably, the quiescent and proliferating RPE-1 and BJ cells revealed the same (in-) sensitivity to ABT-737, proving that ABT-737 (and ABT-263) specifically target senescent cells.

**Figure 2:**
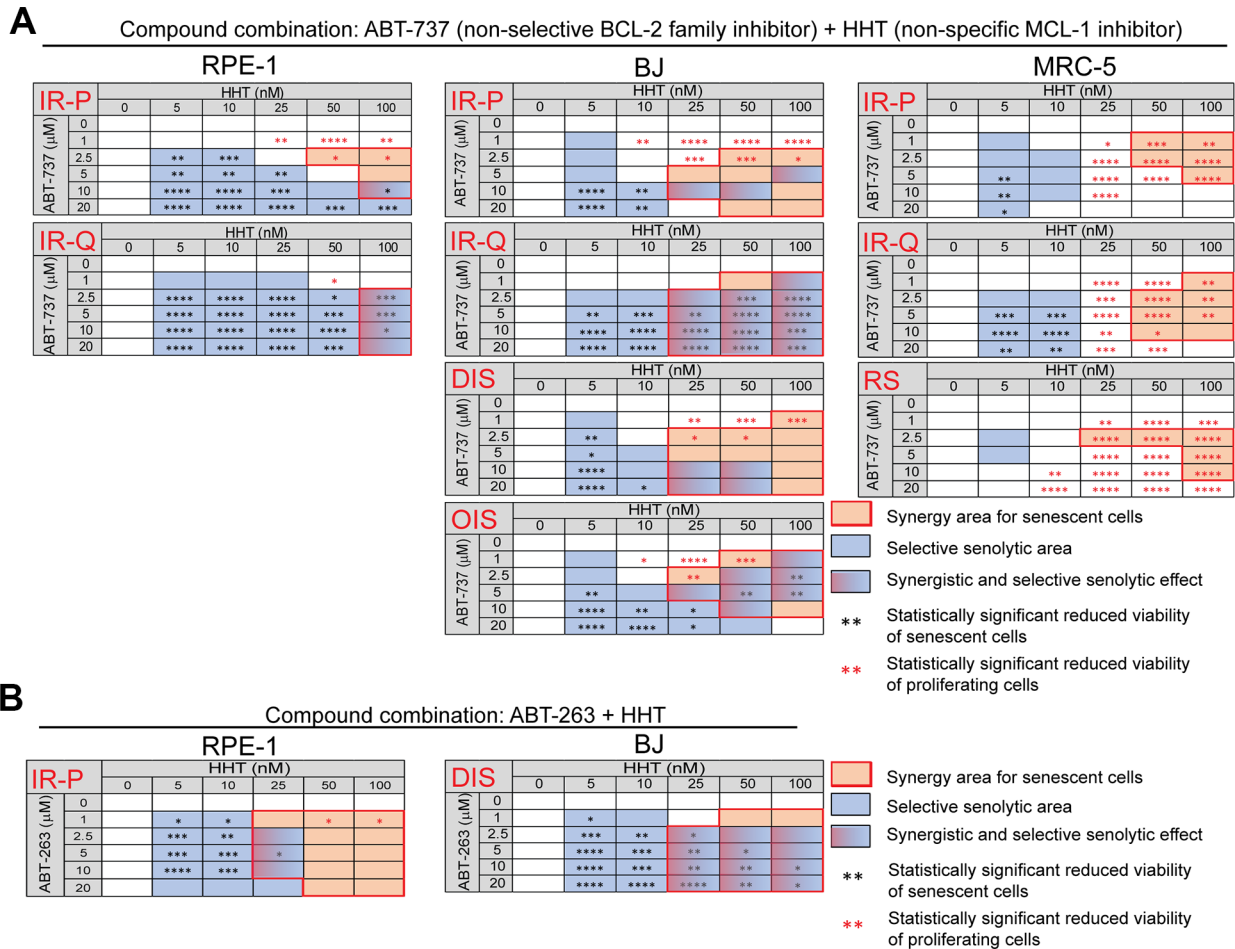
**Homoharringtonine augments the senolytic effects of non-selective BCL-2 inhibitors ABT-737 and ABT-263.** The matrices show the selective senolytic area (blue), synergy area for senescent cells (orange), and the area with synergistic and senolytic effect (blue-orange) of tested compounds. The synergy and senolytic effect of the combination of homoharringtonine (HHT) and ABT-737 (**A**) and HHT and ABT-263 (**B**) on different models of senescence are shown. IR-P – IR-induced senescence derived from proliferating cells; IR-Q – IR-induced senescence derived from quiescent cells; DIS – drug-induced senescence; OIS – oncogene-induced senescence; RS – replicative senescence. The statistical analysis was carried out using the two-tailed Student's t-test; *, P > 0.05; **, P < 0.01; ***, P < 0.001; ****, P < 0.0001. At least three biological replicates were analyzed.

Next, we compared the viability of proliferating, quiescent, and senescent cells exposed to a combination of HHT (0 – 100 nM) with ABT-737 (0 – 20 μM) or ABT-263 (0 – 20 μM) for 24 hours (Figure 2 and Supplementary Figure 2D–2N). We found that HHT potentiated the senolytic effect of ABT-737 mainly in IR-treated quiescent RPE-1 and BJ cells (IR-Q) and oncogene-induced senescent BJ cells (OIS) (Figure 2A). The small area of statistically significant senolytic synergy of HHT and ABT-737 was also detected in IR-treated proliferating RPE-1 (IR-P). In other tested models, IR-P BJ, docetaxel-induced senescent BJ cells (DIS BJ), IR-P MRC-5, IR-Q MRC-5, and replicative senescent MRC-5 cells (RS MRC-5), HHT-potentiating senolytic effect of ABT-737 was not observed. Further, we evaluated whether the HHT-mediated synergistic senolytic effect can be achieved with the BCL-2 family inhibitor ABT-263 (Figure 2B). Indeed, ABT-263 combined with HHT showed the synergistic senolytic effect on both tested senescent models, IR-P RPE-1 and DIS BJ cells. Notably, the synergy of ABT-737/ABT263+HHT cytotoxic effect was observed for all tested proliferating/quiescent models (Supplementary Figure 2D–2N).

Altogether, the combination of HHT with BCL-2 family inhibitors ABT-263 and ABT-737 caused synergistic cytotoxic effects in all tested models. Moreover, HHT significantly increased the senolytic effect of ABT-262 and ABT-737 in a cell type-specific manner.

### Selective inhibition of the MCL-1 augments cytotoxic and senolytic effect of non-selective BCL-2 inhibitors ABT-737 and ABT-263

**Figure 3:**
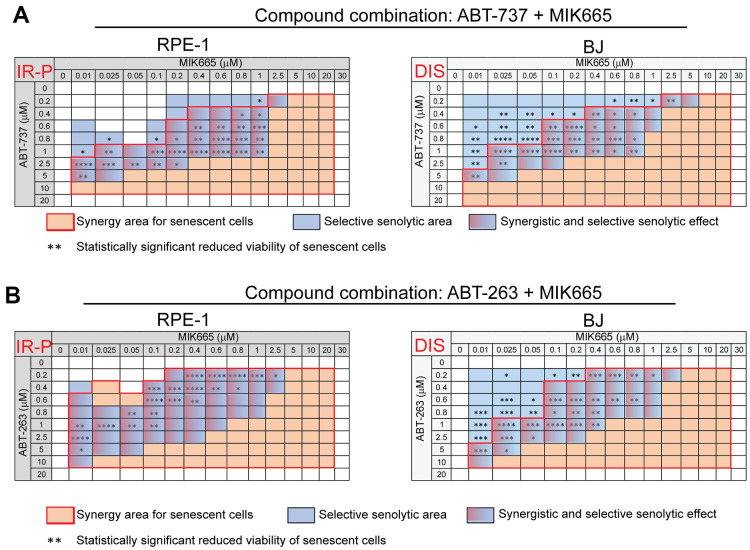
**The selective MCL-1 inhibitor MIK665 augments the senolytic effects of non-selective BCL-2 inhibitors ABT-737 and ABT-263.** The matrices show the selective senolytic area (blue), synergy area for senescent cells (orange), and the area with synergistic and senolytic effect (blue-orange) of tested compounds. The synergy and senolytic effect of the combination of MIK665 and ABT-737 (**A**) and MIK665 and ABT-263 (**B**) on different models of senescence are shown. IR-P – IR-induced senescence derived from proliferating cells; DIS – drug-induced senescence. The statistical analysis was carried out using the two-tailed Student's t-test; *, P > 0.05; **, P < 0.01; ***, P < 0.001; ****, P < 0.0001. At least three biological replicates were analyzed.

As HHT exhibits pleiotropic pharmacologic effects [34, 35], next we tested the impact of a selective MCL-1 inhibitor MIK665 (S64315) [36]. For this purpose, proliferating and senescent IR-P RPE-1 and DIS BJ cells were exposed to MIK665 (0 – 30 μM) separately (Supplementary Figure 3A, 3B) or in combination with ABT-737 (0 – 20 μM; Figure 3A and Supplementary Figure 3C, 3D) or ABT-263 (0 – 20 μM; Figure 3B and Supplementary Figure 3E, 3F) for 24 hours. The analysis of variance (ANOVA) indicated that the sensitivity of senescent and control models to MIK665 used alone differs. Nevertheless, the t-test analysis indicated the IR-P RPE-1 cells were more sensitive compared to control only at one concentration point (20 μM). The significant variance determined for P BJ and DIS BJ is due to the elevated proliferation of P BJ (Supplementary Figure 3A, 3B). Co-treatment of MIK665 with ABT-263 or ABT-737 resulted in an apparent synergistic effect and significantly higher reduction of viability of IR-P RPE-1 and DIS BJ compared to proliferating RPE-1 and BJ populations (Figure 3 and Supplementary Figure 3C, 3E).

Altogether, our data show that a selective MCL-1 inhibitor MIK665 augments the selective senolytic effect of both non-selective BCL-2 inhibitors ABT-263 and ABT-737.

### Senolytic effects of selective BCL-XL and BCL-2 inhibitors combined with the selective MCL-1 inhibitor

As ABT-263 and ABT-737 are non-selective BCL-2 inhibitors [24, 25], further, we tested the cytotoxic and senolytic effects of available selective BCL-2 family inhibitors BCL-2 inhibitor ABT-199 (Venetoclax), BCL-XL inhibitor A1331852, and MCL-1 inhibitor S63845, either alone or in combination. First, proliferating and senescent RPE-1 cells were exposed to ABT-199 (0 – 100 μM) and A1331852 (0 – 100 μM) alone (Supplementary Figure 4A, 4B). In agreement with a previous study [37], BCL-XL inhibitor A1331852 showed an apparent senolytic effect on irradiated RPE-1 senescent cells. Surprisingly, at high concentrations, ABT-199 demonstrated a marked senolytic effect as well, in contrast to previous findings [24, 25]. Note that the MCL-1 inhibitor S63845 tested alone did not show any senolytic effect in a concentration range of 0 – 50 μM. Contrarily, the proliferation cells were even more sensitive to 20 and 30 μM concentrations of S63845 (Supplementary Figure 4C).

**Figure 4:**
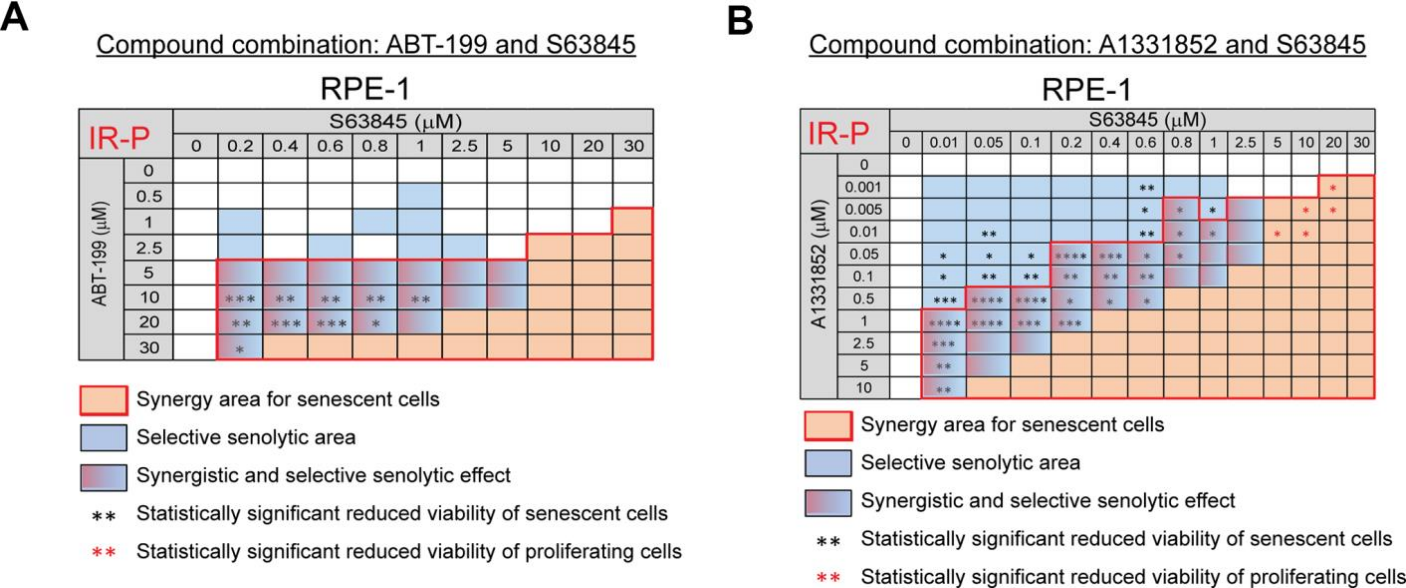
**The selective MCL-1 inhibitor S63845 augments the senolytic effects of selective BCL-2 (ABT-199) and BCL-XL (A1331852) inhibitors.** The matrices show the selective senolytic area (blue), synergy area for senescent cells (orange), and the area with synergistic and senolytic effect (blue-orange) of tested compounds. The synergy and senolytic effect of the combination of ABT-199 and S63845 (**A**) and A1331852 and S63845 (**B**) on the model of IR-induced senescence derived from proliferating cells (IR-P). The statistical analysis was carried out using the two-tailed Student's t-test; *, P > 0.05; **, P < 0.01; ***, P < 0.001; ****, P < 0.0001. At least three biological replicates were analyzed.

Next, we analyzed the combination of BCL-2 inhibitor ABT-199 (0 – 30 μM) with MCL-1 inhibitor S63845 (0 – 30 μM). We observed a significant synergistic effect of this combination together and an apparent selective senolytic effect on IR-P RPE-1 cells in a broad concentration range (Figure 4A and Supplementary Figure 4D). Similarly, substituting BCL-2 inhibitor ABT-199 for BCL-XL inhibitor A1331852 (0 – 10 μM) in combination with MCL-1 inhibitor S63845 (0 – 30 μM) led to an even more robust synergistic and selective senolytic effect (Figure 4B and Supplementary Figure 4E).

In summary, our data show that the MCL-1 inhibitor S63845 synergistically augments the senolytic effect of the selective BCL-2 inhibitor ABT-199 and the selective BCL-XL inhibitor A1331852. Moreover, we found that the selective BCL-2 inhibitor ABT-199, used clinically, can exert senolytic effects.

### BCL-2 inhibitor-resistant cells harbor elevated MCL-1

**Figure 5:**
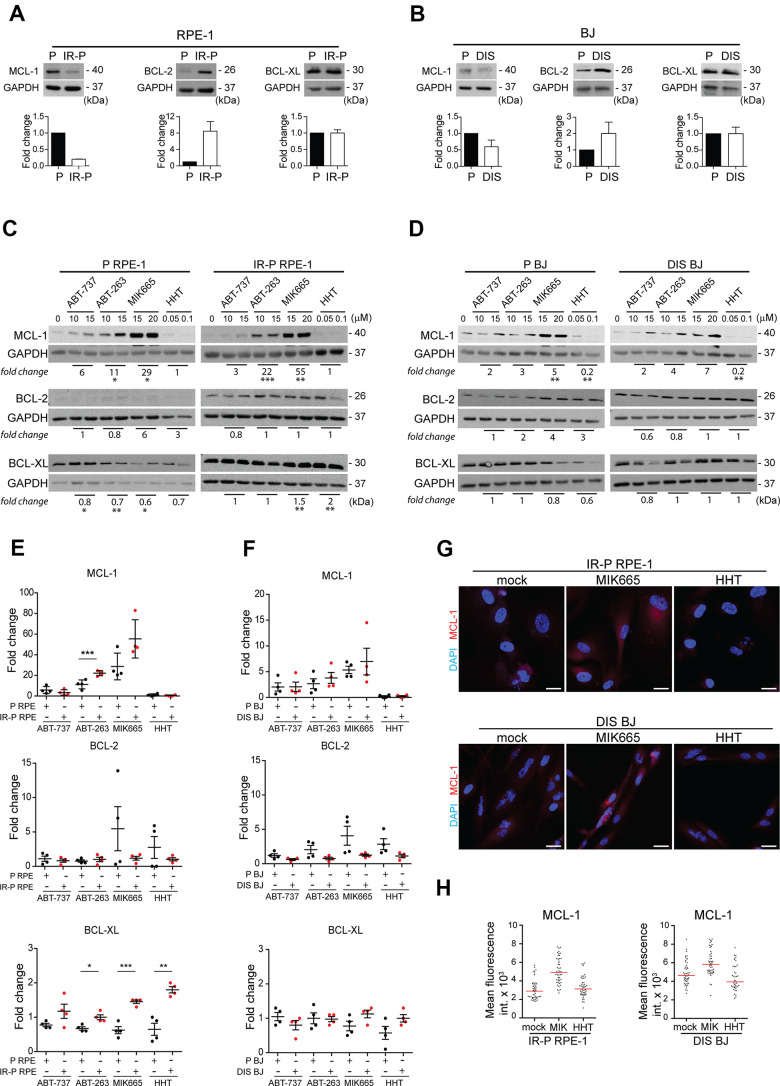
**Analysis of the expression levels of the anti-apoptotic proteins in the cell populations resistant to ABT-263, ABT-737, MIK665, and HHT.** Immunoblotting analysis of MCL-1, BCL-2, and BCL-XL anti-apoptotic protein levels in proliferating (P) and senescent cells induced by (**A**) IR (IR-P RPE) or (**B**) docetaxel (DIS BJ). The difference between the levels detected in P and IR-P or DIS cells is expressed as fold-change. Three independent experiments were analyzed. Immunoblotting analysis of MCL-1, BCL-2, and BCL-XL anti-apoptotic protein levels in (**C**) RPE-1 (P and IR-P) and (**D**) BJ (P and DIS) after 24-h long exposure to ABT-263, ABT-737, MIK665, and HHT. The difference between the untreated control and the 'resistant' population was expressed as the mean of fold change obtained for two concentrations in two independent experiments. (**E**, **F**) Quantitative analysis of immunoblots (presented in **C** and **D**) comparing the fold-change of anti-apoptotic protein levels in treatment-surviving populations between proliferating and senescent cells. The mean ± SD is shown. All statistical analyses were carried out using the two-tailed Student's t-test; *, P > 0.05; **, P < 0.01; ***, P < 0.001. (**G**) Indirect immunofluorescence staining for MCL-1 levels (red signal) in senescent IR-RPE-1 and DIS-BJ cells after treatment with MIK665 (15 μM) and HHT (100 nM). Cell nuclei were stained by DAPI (blue signal). (**H**) Quantitative analysis of MCL-1 levels in individual cells (presented in **G**), expressed as a mean fluorescence intensity in cytoplasm of individual cells (n > 40). Two different regions of each individual cell were analyzed and their mean plotted. Note the heterogeneous MCL-1 expression represented as a variance of fluorescence signal. Bar, 30 μm.

To examine the mechanism of cooperation between BCL-2 inhibitors, first, we determined the levels of MCL-1, BCL-2, and BCL-XL proteins in proliferating and senescent RPE-1 and BJ cells by immunoblotting (Figure 5A, 5B). As expected [24], senescent cells showed a higher level of BCL-2 than proliferating cells, whereas the level of MCL-1 was lower in senescent IR-P RPE-1 and DIS BJ cells (Figure 5A, 5B). No differences in BCL-XL levels were detected in proliferating and senescent RPE-1 and BJ cells (Figure 5A, 5B).

Next, we examined the nature of the synergy between BCL-2 family inhibitors (ABT-737 and ABT-263) and MCL-1 inhibitors (MIK665 and HHT). We tested the notion that the cell population is composed of cells with different sensitivity to ABT-737, ABT-263, MIK665, or HHT. For this purpose, we exposed proliferating and senescent (IR-P-RPE-1 and DIS-BJ) cells to each compound separately using two concentrations close to 50% cell viability in the most sensitive cell type (i.e., 10 and 15 μM for ABT-737 or ABT-263, 15 and 20 μM for MIK655, 50 and 100 nM for HHT) for 24 hours. The surviving adherent cells were harvested for immunoblotting to determine the level of the inhibitor’s targets, MCL-1, BCL-2, and BCL-XL. As shown in Figure 5C, 5D, the RPE-1 and BJ proliferating and senescent cells surviving the treatment of ABT-737, ABT-263, or MIK665 showed a highly elevated level of anti-apoptotic MCL-1 protein compared to untreated cells. In the same line, the RPE-1 cells (P and IR-P) resistant to the HHT had an unchanged level of MCL-1, whereas the resistant BJ cells (P and DIS) manifested a significantly lower level than the untreated control. Moreover, the population of the RPE-1 senescent cells surviving the treatment with MIK665 and HHT harbored significantly elevated levels of BCL-XL. Oppositely, the MIK665- and HHT-resistant population of proliferating cells (P RPE-1) exerted a significantly lower level of this anti-apoptotic protein compared to control cells. The levels of BCL-2 were not changed in any resistant population analyzed.

This experiment revealed that single-compound treatment with ABT-737, ABT-263, HHT, and MIK665, respectively, was performed on resistant populations that were heterogeneous and encompassed cells with different levels of anti-apoptotic proteins. Therefore, we asked whether such variability could explain the specific senolytic effect observed after treatment with compound combinations. Based on immunoblotting data (Figure 5C, 5D), we compared the fold change of MCL-1, BCL-XL, and BCL-2 levels between resistant senescent and proliferating populations (Figure 5E, 5F). This analysis revealed a significantly higher level of MCL-1 in senescent RPE-1 cells surviving the ABT-263 treatment (Figure 5E). Interestingly, the level of BCL-XL was significantly higher in senescent RPE-1 cells resistant to all tested treatments except ABT-737 compared to proliferating cells (Figure 5E). No substantial changes were detected for BCL-2 (Figure 5E). Importantly, we identified similar tendencies in the levels of MCL-1, BCL-XL, and BCL-2 in BJ cells (proliferating and senescent) surviving all tested treatments (Figure 5F). As shown in Figure 5G, 5H, the levels of MCL-1 indeed differed among individual senescent cells (IR-P RPE-1 and DIS BJ), indicating heterogeneous expression of MCL-1 in the initial cell populations. In agreement with immunoblotting data, the MCL-1 fluorescence signal increased after exposure to MIK665 in both senescent RPE-1 and BJ cells and decreased after HHT treatment in senescent BJ cells (Figure 5H).

**Figure 6:**
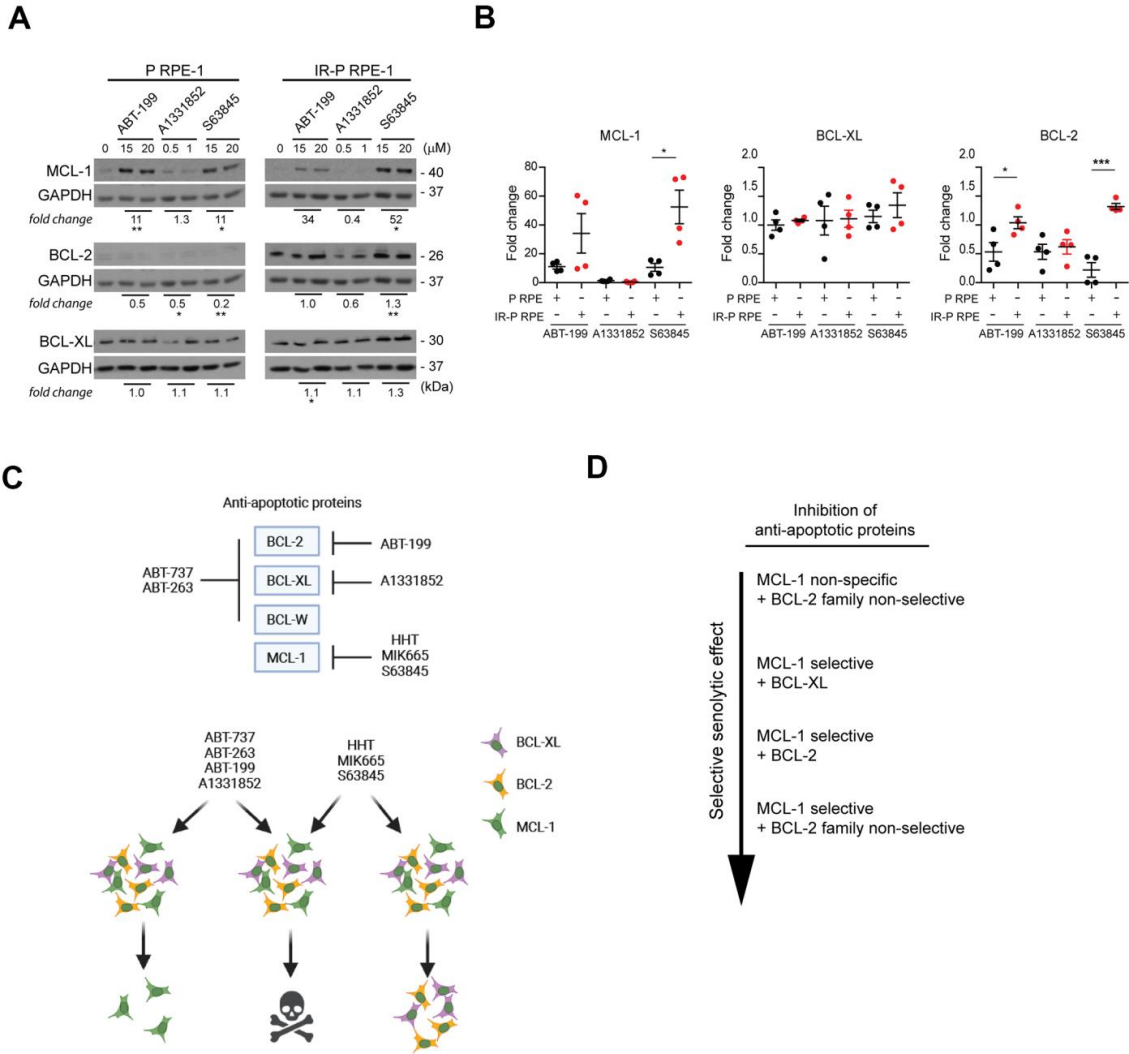
**Analysis of the expression levels of the anti-apoptotic proteins in the cell populations resistant to ABT-199, A1331852, and S63845.** (**A**) Immunoblotting analysis of MCL-1, BCL-2, and BCL-XL anti-apoptotic protein levels in proliferating (P) and IR-induced senescent (IR-P RPE) cells after treatment with ABT-199, A1331852, and S63845. (**B**) The quantitative analysis of immunoblots (presented in **A**) comparing the fold-change of anti-apoptotic protein levels in treatment-surviving populations between proliferating and senescent cells. Two independent experiments were analyzed. The mean ± SD is shown. All statistical analyses were carried out using the two-tailed Student's t-test; *, P > 0.05; **, P < 0.01; ***, P < 0.001. (**C**) BCL-2 protein family targets of inhibitors analyzed (upper panel). Schematic representation of the mechanism of synergy among BH3 mimetics and MCL-1 inhibitors (lower panel). (**D**) Inhibitor combinations arranged according to the effectiveness of the selective senolytic effect.

To further support this notion, we analyzed the levels of BCL-2 proteins surviving the exposure to BCL-2-inhibitor ABT-199, BCL-XL inhibitor A1331852, and MCL-1 inhibitor S63845. Proliferating (P RPE-1) and IR-senescent RPE-1 (IR-P RPE-1) cells were exposed to individual inhibitors at doses close to IC50 (Figure 6A, 6B). MCL-1 was significantly elevated both in proliferating and senescent RPE-1 cells exposed to ABT-199 and S63845. The levels of BCL-2 tend to decrease in surviving proliferating and remained unchanged in senescent RPE-1 cells for all three inhibitors. BCL-XL levels remained unchanged in all conditions. Next, we compared the fold change of MCL-1, BCL-XL, and BCL-2 levels between the ABT-199-, A1331852-, and S63845-resistant senescent and proliferating populations (Figure 6B). Again, this analysis revealed a significantly higher levels of MCL-1 and BCL-2 in senescent RPE-1 cells surviving the ABT-199 and S63845 treatment. No substantial changes were detected for BCL-XL.

In summary, our findings indicate that the cell populations resistant to ABT-263, ABT737, ABT-199, and S63845 harbor higher levels of MCL-1 protein. Besides, the senescent RPE-1 population less sensitive to HHT or MIK665 and ABT-199 or S63845 contains an elevated BCL-XL and BCL-2, respectively. These observations could explain the synergic senolytic effect of non-selective BCL-2 and MCL-1 inhibitors due to simultaneously targeting subsets of cells with heterogeneous BCL-2 protein expression within the initial total senescent cell populations.

## DISCUSSION

The accumulation of senescent cells over time is thought to affect normal tissue function and contribute to aging and the development of age-associated diseases [38–40]. These pathogenic effects are likely mediated by a broad spectrum of pro-inflammatory cytokines, chemokines, growth factors, and extracellular matrix proteins secreted by senescent cells, commonly referred to as the senescence-associated secretory phenotype (SASP). The SASP factors induce mild but chronic inflammation and alter the local tissue environment, provided senescent cells are not cleared by the immune system [38–42]. In addition to bypassing the immune system, the accumulation and persistence of senescent cells in tissues are associated with their resistance to extrinsic and intrinsic pro-apoptotic stimuli [18, 43] executed mainly by members of the BCL-2 protein family [20, 21]. Consistent with such notion, the genetically engineered or drug-mediated clearance of senescent cells in mice increases the health span, delaying the onset of several age-associated diseases in both progeroid and naturally aged animals [10, 11, 13, 24, 44–48].

Unfortunately, senolytic therapy with BCL-2 inhibitors is not void of adverse side effects. For example, treatment with the BCL-2 inhibitor and a senolytic drug ABT-263 causes transient thrombocytopenia [49–51], which may reflect the fact that mature megakaryocytes themselves appear to be physiologically senescent cells [52]. Therefore, current efforts focus on identification of drug combinations with synergistic senolytic effects in order to reduce drug toxicity and adverse therapeutic effects in monotherapy. For instance, the above-mentioned BCL-2 inhibitor ABT-263 was tested in combination with the senolytic drug piperlongumine [53].

In this study, with the goal of decreasing the toxicity and potential onset of resistance to senolytic BCL-2 inhibitor monotherapy, we explored the effects of combined treatment covering both BCL-2 and MCL-1 anti-apoptotic factors in human cells. Senolytic and senomorphic compounds show promise for potential applications in treating several disorders. Therefore, there is an urgent, unmet need to develop senolytic therapies specific to diverse conditions. To cover various scenarios, we utilized *in vitro* cell models representing four prototypical modes of cellular senescence to test the synergy of BCL-2 inhibitors – replicative, oncogene-, radiation-, and drug-induced.

Replicative senescence corresponds to 'natural' exhaustion of replicative potential by clonal selection of cell clones adapting best to *in vitro* cell culture conditions [54, 55] and halted in cell cycle progression predominantly by DNA damage-activated cell cycle checkpoints in response to DNA damage caused by telomere attrition [56]. There is a hope that the administration of specific senolytics targeting these cells throughout life may delay or even avoid the onset of several aging-associated diseases caused by accumulating senescent cells, as well as postpone the process of aging itself [57]. Cellular senescence induced by activated oncogenes [58, 59] resulting in development of benign preneoplastic lesions (see, e.g., ref. [60]) is presumed to act as primary tumorigenesis barrier [61]. Removal of preneoplastic lesions by senolytics is believed to lessen the risk of malignant transformation [62].

Last but not least, radio- and chemo-therapy trigger cellular senescence and senescence resembling features in both normal cells (including tumor-associated stromal cells; reviewed, e.g., in ref. [63]) and senescence-like phenotype in tumor cells (see, e.g., ref. [64]). Therefore, there is a potential for beneficial application of senolytics as components of adjuvant therapy strategies to negate the adverse effects of the standard-of-care antiproliferative therapeutic approaches.

As drug repurposing is economically much more favorable than developing new drugs [65], we focused on combinations of already clinically approved drugs used in the medication of neoplastic diseases. Initially, we tested protein synthesis and MCL-1 non-specific inhibitor homoharringtonine [32, 66], with the benefit of its clinical use to treat chronic myeloid leukemia with only minor side effects [33]. HHT itself was, in general, cytotoxic without a specific senolytic effect. In combination with BCL-2 inhibitors ABT-737 and ABT-263, HHT showed additive cytotoxicity on proliferating, quiescent, and senescent cells. Nevertheless, in a narrow window of concentrations, HHT markedly potentiated the cytotoxic effect of ABT-737 on radiation-induced senescent RPE-1 cells, indicating that MCL-1 inhibitors possess the promising potential to augment the senolytic effects of BCL-2 inhibitors. To further validate this notion, we chose a more selective MCL-1 inhibitor, MIK665 (S64315) [36]. Indeed, MIK665, besides its indiscriminate cytotoxic effect when used alone, robustly synergized with the senolytic effects of ABT-263 and ABT-737, respectively. This effect was particularly apparent when using ionizing radiation- and docetaxel-induced senescent RPE-1 and BJ cells, further supporting the concept that the combination of BCL-2 and MCL-1 inhibitors could be beneficial in senolytic therapy.

We extended this observation by testing combinations of other selective BCL-2 inhibitors. Notably, BCL-2 inhibitor ABT-199 (Venetoclax) with MCL-1 inhibitor S63845 or BCL-XL inhibitor A1331852 with MCL-1 inhibitor S63845 showed marked selective senolytic effect at doses more than 20 times lower than those necessary to show a similar effect when used individually. It should be emphasized that this compound under the brand name Venclexta and Venclyxto successfully passed several clinical studies and is used to treat acute myeloid leukemia, chronic lymphocytic leukemia, and small lymphocytic lymphoma [67, 68]. Yosef et al. reported the senolytic effect of ABT-199 on OIS but not RS and DIS [24]. In concordance with our results demonstrating the selective senolytic effect on irradiated RPE-1 cells, a new study showing the selective senolytic effect of Venetoclax on radiotherapy-induced senescent sarcoma cell lines has been published [69], broadening the potential application range of Venetoclax. It should be noted that the combination of BCL-2 (Venetoclax) and MCL-1 (S63845) or BCL-XL (A-1331852) was effective against precursor B-cell acute lymphoblastic leukemia, as reported recently using patients' xenografts in mouse model [70].

In an attempt to elucidate the mechanistic basis of the enhanced senolytic effect of combined BCL-2 and MCL-1 inhibitors, our present work revealed that the surviving proliferating and radiation-induced senescent RPE-1 cells exposed to the concentrations of ABT-263, MIK665, ABT-199, and S63845 inhibitors close to IC50 harbor a higher level of MCL-1 protein than the non-treated cells, thereby indicating heterogeneity within the initial cell population concerning the MCL-1 protein level. Indeed, a closer examination of the parental population of senescent cells disclosed a large variation of MCL-1 protein levels among the individual cells. In addition, cells surviving the treatment with the BCL-2 inhibitor ABT-199 harbor higher levels of BCL-2 protein.

On the other hand, radiation-induced senescent RPE-1 cells surviving the treatment with both MCL-1 inhibitors HHT and MIK665 express higher levels of BCL-XL protein, indicating the mechanism of cross-resistance to BCL-2 and MCL-1 inhibitors (see scheme in Figure 6C). Moreover, the cells resistant to HHT showed a lower level of MCL-1, which agrees with the previous report [32]. Similar, though not so evident effects were observed in proliferating and docetaxel-induced senescent BJ cells suggesting that enhanced sensitivity to this drug combination likely reflects an analogous mechanism.

In conclusion, we propose that the selective senolytic effect of non-MCL-1 BCL-2 family inhibitors such as the known senolytics ABT-263 can be augmented by concomitant treatments with selective MCL-1 inhibitors. The mechanistic basis of this synergy reflects, at least in part, the heterogeneous expression of individual members of the BCL-2 protein family among the phenotypically heterogeneous senescent cells (Figure 6D). Therefore, combining two BCL-2 inhibitors targeting different members of the BCL-2 family could surmount this heterogeneity, decrease the resistance of senescent cells to apoptosis and thus enhance the selective senolytic effect. Another potential advantage of combining synergistically acting senolytic compounds could be an opportunity to use lower drug doses and thus diminish overall toxicity. Further work is needed to validate the effectiveness of BCL-2 inhibitor combinations *in vivo*.

## MATERIALS AND METHODS

### Cell culture

Human telomerase-immortalized retinal pigment epithelial cells (RPE-1, ATCC® CRL-4000™) were cultured in Dulbecco’s modified Eagle’s medium (DMEM, Thermo Fisher Scientific, Waltham, MA, USA) with high glucose (4.5 g/L). Primary human skin (BJ, ATCC® CRL-2522™, population doublings 25) and embryonal lung fibroblasts (MRC-5, ATCC® CCL-171™, population doublings 24 – 52) were cultured in Dulbecco’s modified Eagle’s medium (DMEM, Thermo Fisher Scientific, Waltham, MA, USA) with low glucose (1 g/L). Both cell culture media were supplemented with 10% fetal bovine serum (FBS, Gibco/Thermo Fisher Scientific) and non-essential amino acids (NEAA), 100 units/mL of penicillin, and 100 μg/mL of streptomycin (Sigma-Aldrich). Cells were kept at 37° C under a 5% CO_2_ atmosphere and 95% humidity.

### Antibodies

The following primary and secondary antibodies were used for immunoblotting: mouse monoclonal anti-MCL-1 (clone 10, sc-12756) purchased from Santa Cruz Biotechnology, Inc., Dallas, TX, USA; rabbit monoclonal anti-BCL-2 (clone 50E3, #2870), rabbit monoclonal anti-BCL-XL (clone 54H6, #2764) purchased from Cell Signaling Technology, Inc., Danvers MA, USA; and mouse monoclonal anti-GAPDH (GTX3066) purchased from GeneTex, Inc., Irvine, CA, USA; goat anti-mouse (170-6516) or anti-rabbit IgG (H + L)-HRP conjugate (170-6515) purchased from BioRad, Hercules, CA, USA. For indirect immunofluorescence, mouse monoclonal anti-MCL-1 (clone 10, sc-12756) purchased from Santa Cruz Biotechnology, Inc., Dallas, TX, USA and Alexa Fluor 568 goat anti-mouse (A-11031) secondary antibody obtained from Invitrogen/Thermo Fisher Scientific, Waltham, MA, USA, were used.

### Ionizing radiation-induced cellular senescence

Proliferating or contact-inhibited (quiescent) cells were exposed to a single dose of X-rays using Pantak HF160 (Gulmay, Surrey, UK) X-ray instrument equipped with Pantak Seifert HF320 generator, MXR-161 X-ray tube (Comet AG, Flamatt, Switzerland), and an aluminum filter using current 1 – 10 mA to obtain ionizing radiation (IR)-induced senescent cells. The 10 Gy was a sufficient dose to trigger stable cellular senescence in BJ and MRC-5 cells. However, 20 Gy was necessary to avoid a bypass of senescence in RPE-1, as referred to in our previous article [71]. After irradiation, the cells were cultured for an additional 7 (MRC-5 and RPE-1) or 21 (BJ) days until the development of the senescent phenotype.

### Drug-induced cellular senescence

BJ cells were exposed to 2 nM docetaxel for 7 days to induce drug-induced senescence (DIS), as described previously [72].

### Oncogene-induced cellular senescence

To prepare oncogene-induced senescent cells (OIS), BJ cells carrying a tetracycline-regulated ectopic expression of H-RasV^12^ oncogenic mutant [73, 74] were exposed to 2 μg/mL of doxycycline every 48 hours for 14 days until loss of proliferative activity and development of senescent phenotype.

### Replicative cellular senescence

MRC-5 cells were regularly split in a 1 : 2 ratio until the cell division ceased (population doubling 52) to bring MRC-5 cells to replicative senescence (RS).

### Senescence-associated beta-galactosidase activity

Cells were fixed with 0.5% glutaraldehyde at room temperature for 15 min. After that, cells were washed twice with 1 mM MgCl_2_/PBS and incubated with X-Gal staining solution at 37° C for 3 hours. The staining was terminated by three consecutive washes with ddH_2_O. Finally, the cells were let dry, mounted with Mowiol containing DAPI and imaged on the Leica DM6000 fluorescent microscope using the HC PLAN APO 20×/0.70 DRY PH2 objective and color CCD camera Leica DFC490 (Leica Microsystems GmbH, Wetzlar, Germany).

### DNA replication assay

Cells were incubated with 10 μM 5-ethynyl-2’-deoxyuridine (EdU) for 24 hours and fixed with 4% formaldehyde at room temperature for 15 min. To visualize EdU incorporation, click chemistry was performed with Click-iT™ EdU Cell Proliferation Kit with Alexa Fluor™ 647 dye (Thermo Fisher Scientific, Waltham, MA, USA) according to manufacturer’s instructions. The stained cells were acquired by high-content imaging using inverted wide-field microscope (Olympus IX81) equipped with UPLXAPO 20×/0.8 DRY CORR; FWD 0.6; CG 0 – 2 objective and sCMOS camera Hamamatsu ORCA-Flash4.0 LT+90, 6.5 μm pixel.

### Cell viability assay

To determine cell viability by the crystal violet assay [75], cells were plated in 96-well plates at densities indicated in Figure 1A. Cells were exposed to 0.2 to 20 μM ABT-737 (Selleckchem, S1002) and ABT-263 (Selleckchem, S1001), 5 to 100 nM homoharringtonine (HHT, Sigma, SML1091) and 0.01 to 30 μM MIK665 (S64315, Chemietek, CT-MIK665) for 24 hours. Then the cells were washed twice with 150 μL PBS and stained with 30 μL 0.5% w/v crystal violet in 20% methanol for 15 minutes. Next, the plates were washed 5 times with ddH_2_O and left to dry overnight. Crystal violet was solubilized with 75 μL 0.2% Triton X-100 (Sigma) in PBS for 15 minutes. The absorbance was read at 595 nm using a microplate reader (Multiskan EX, Thermo Electron Corporation). Each condition was measured at least in triplicate, and the absorbance of crystal violet in treated cells was expressed as a percentage of absorbance in untreated cells.

### SDS-PAGE and immunoblotting

Cells were washed with PBS, harvested into SDS sample lysis buffer (SLB; 2% SDS, 63 mM Tris-HCl, pH 6.8, 10% glycerol), sonicated, and centrifuged. Protein concentration was determined by BCA (Pierce Biotechnology, IL, Rockford, USA), samples adjusted to equal protein amount (40 μg) with SLB containing DTT (1% final conc.) and bromophenol blue (0.02% final conc.) and separated by SDS–PAGE in Tris-glycine-SDS buffer (BioRad, 1610772). Proteins were electrotransferred onto a nitrocellulose membrane (0.45 μm NC, Amersham™, GE Healthcare Life Sciences) using wet transfer in Tris-glycine buffer (BioRad, 1810704) with 10% methanol (Sigma, 59304) and after blocking with 5% non-fat milk in PBS/Tween-20 were detected using specific antibodies and horseradish peroxidase (HRP)-conjugated secondary antibodies. Peroxidase activity was detected by ECL detection reagents (Thermo Fisher Scientific, Waltham, MA, USA). Quantitative analysis was done by Image J 1.48v program with GAPDH as the internal control.

### Indirect immunofluorescence

Cells grown on glass coverslips were rinsed with PBS, fixed with mixture of 3% formaldehyde and 0.2% glutaraldehyde in PBS (30 min on ice), washed three times with 0.2% BSA in PBS, blocked with 10% FBS and 0.2% saponin in PBS (30 min at RT), and incubated with primary antibody diluted in blocking solution (18 hours at 4° C). After that the cells were washed three times with 0.2% saponin in PBS and incubated with secondary antibody. Subsequently, cells were counterstained with 1 μg/mL DAPI for 4 min, washed three times with PBS for 5 min, let dry and mounted with Antifade Pro-long Gold Mounting Media. The wide-field images were subsequently acquired on the Leica DM6000 fluorescent microscope using the HC PLAN APO 20×/0.70 DRY PH2; FWD 0.59; CG 0.17 objective and Leica DFC 9000 – monochromatic sCMOS camera; 6.5 μm pixel, QE: min. 82%.

### Data processing and statistical analysis

Graphs were generated using GraphPad Prism 5.04 (GraphPad Software, La Jolla, CA, USA). The data are expressed as the mean ± S.D. from three independent experiments. Statistical differences for the two groups were analyzed by two-tailed Student’s t-test.

## Supplementary Material

Supplementary Figures
