# Supplementary material for: Synergism of BCL-2 family inhibitors facilitates selective elimination of senescent cells
Source: Aging (Albany NY). 2022 Aug 8;14(16):6381–414. doi: 10.18632/aging.204207 (PMC9467395; doi:10.18632/aging.204207)
Supplement: Supplementary Figures [file aging-14-204207-s001.pdf]

SUPPLEMENTARY FIGURES

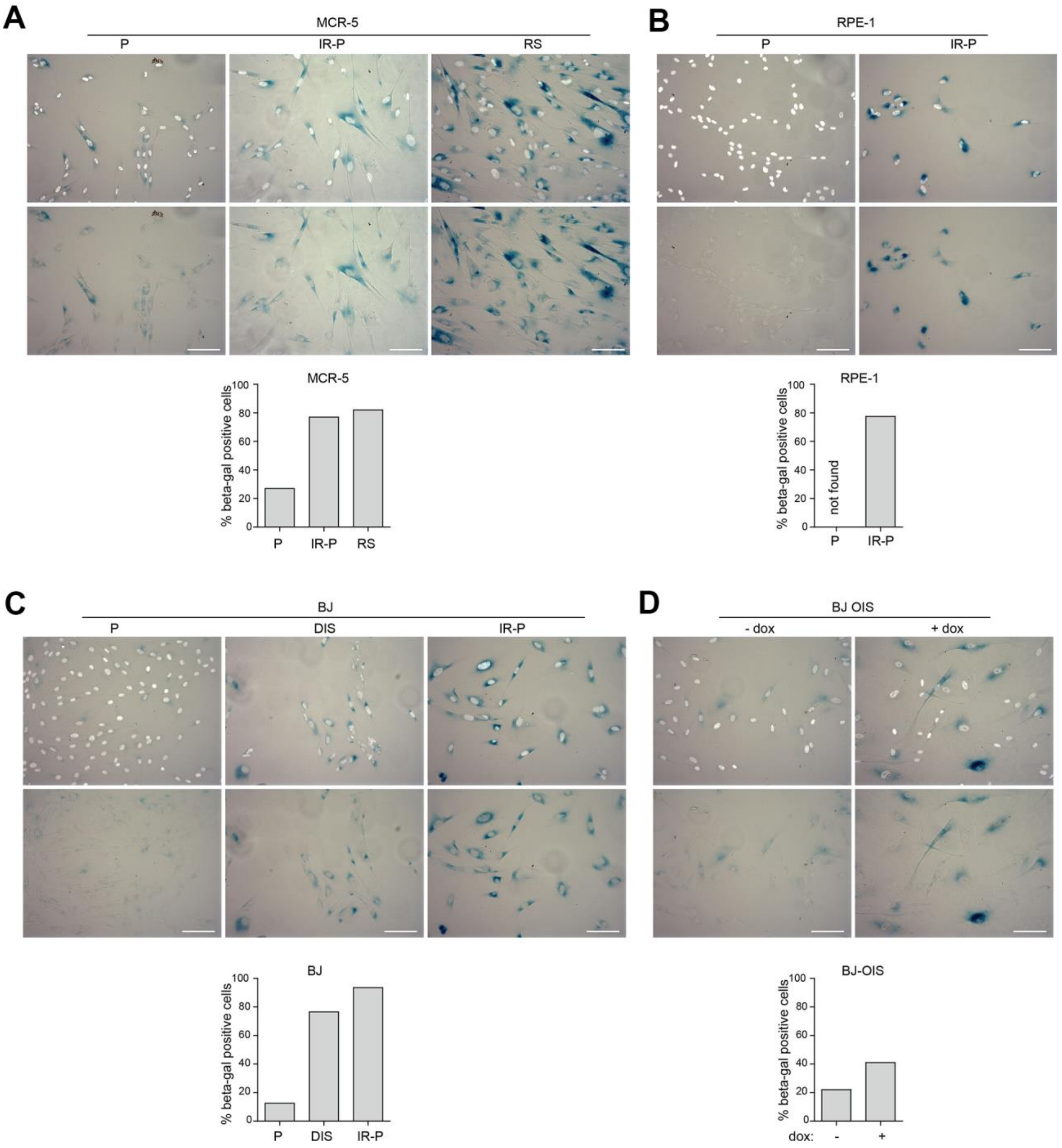

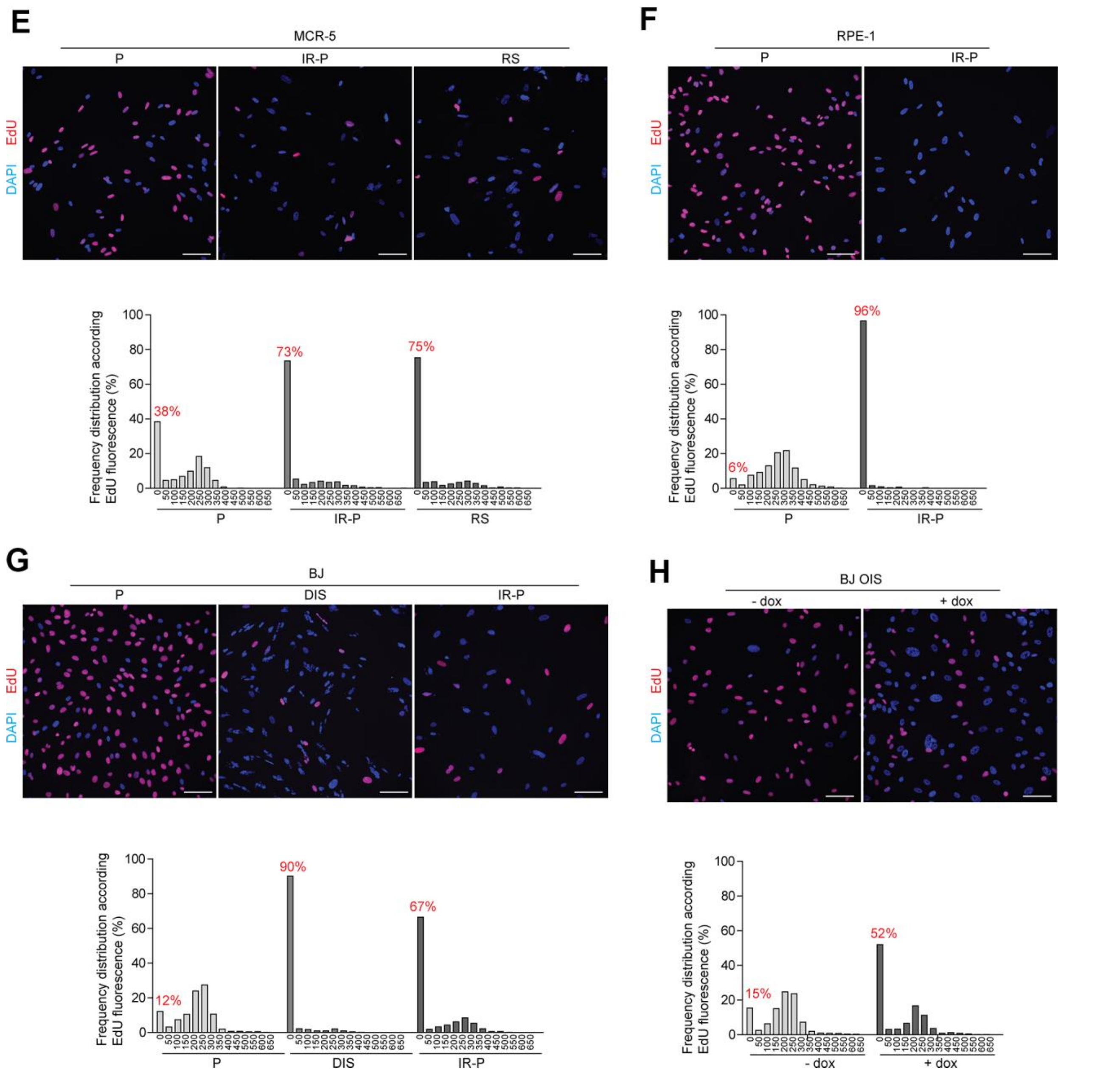

Supplementary Figure 1. Determination of cellular senescence markers in models of senescence. Senescence-associated β-galactosidase activity in (**A**) replicative (RS) and irradiated (10 Gy; IR-P) senescent MRC-5, (**B**) irradiated RPE-1 (20 Gy; IR-P), (**C**) docetaxel-induced BJ (BJ DIS), irradiated BJ (10 Gy; BJ IR-P), and (**D**) oncogenic Ras-induced BJ (BJ OIS; after induction with doxycycline, +dox) cells. Proliferating cells (-dox for BJ OIS) were used as controls. Cell nuclei were stained by DAPI (upper row; white color). The percentage of β-galactosidase-positive cells in proliferating and senescent populations was plotted (n > 100). DNA replication activity detected by EdU incorporation (red color) in (**E**) replicative (RS) and irradiated (10 Gy; IR-P) senescent MRC-5, (**F**) irradiated RPE-1 (20 Gy; IR-P), (**G**) docetaxel-induced BJ (BJ DIS), irradiated BJ (10 Gy; BJ IR-P), and (**H**) oncogenic Ras-induced BJ (BJ OIS; after induction with doxycycline, +dox) cells. Proliferating cells (-dox for BJ OIS) were used as controls. Cell nuclei were stained by DAPI (blue color). The percentage of EdU-positive cells was plotted (n > 100). Bar, 30 µm.

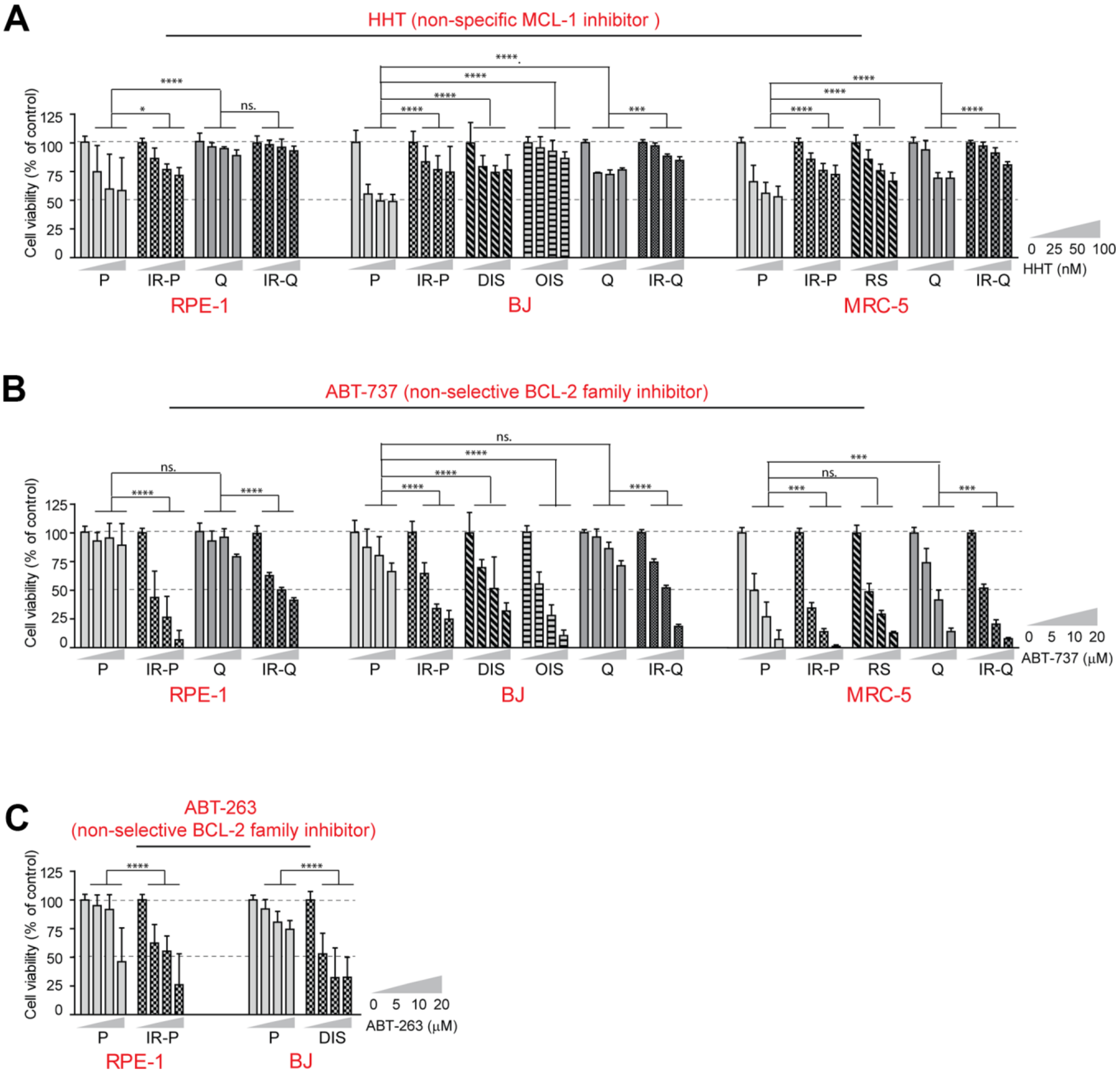
A
HHT (non-specific MCL-1 inhibitor)
Cell viability (% of control)
0 25 50 100
HHT (nM)
P
IR-P
Q
IR-Q
DIS
OIS
RS
RPE-1
BJ
MRC-5
B
ABT-737 (non-selective BCL-2 family inhibitor)
0 5 10 20
ABT-737 (μM)
C
ABT-263
(non-selective BCL-2 family inhibitor)
0 5 10 20
ABT-263 (μM)

D

RPE-1: IR-induced senescence (from proliferation)

Compound combination: ABT-737 and HHT

Proliferating

P RPE-1		HHT (nM)					
		0	5	10	25	50	100
ABT-737 (µM)	0	100	95	97	75	60	59
	1	102	95	91	67	50	47
	2.5	97	97	94	66	46	36
	5	93	89	89	64	38	28
	10	96	89	82	54	26	16
	20	89	83	80	50	17	9

Cell viability (%): 100, 75, 50, 25, 0

Senescent

IR-P RPE-1		HHT (nM)					
		0	5	10	25	50	100
ABT-737 (µM)	0	100	92	88	86	77	72
	1	92	97	88	83	75	68
	2.5	72	78	76	70	58	45
	5	44	54	54	45	38	25
	10	26	19	20	20	18	6
	20	7	4	5	6	6	1

Synergy scoring (HSA) → **Synergy map**

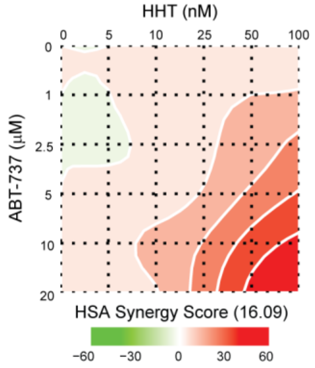

Difference between viability of proliferating and senescent cells → **Selective senolytic effect**

		HHT (nM)					
		0	5	10	25	50	100
ABT-737 (µM)	0						
	1				**	****	**
	2.5		**	***		*	*
	5		**	**	**		
	10		****	****	***		*
	20		****	****	****	***	***

Synergy area for senescent cells

Selective senolytic area

Synergistic and selective senolytic effect

** Statistically significant reduced viability of senescent cells

** Statistically significant reduced viability of proliferating cells

Synergy scoring (HSA) → **Synergy map**

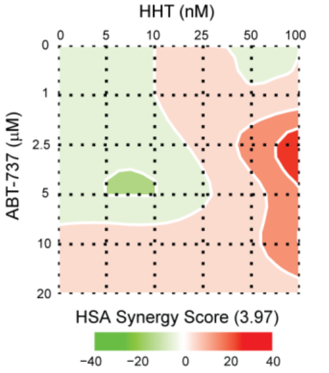

E

RPE-1: IR-induced senescence (from quiescence)

Compound combination: ABT-737 and HHT

Quiescent

Q RPE-1		HHT (nM)					
		0	5	10	25	50	100
ABT-737 (µM)	0	101	102	103	96	95	89
	1	94	97	97	96	86	82
	2.5	100	102	100	100	89	75
	5	93	95	95	94	78	57
	10	96	99	94	90	59	38
	20	79	85	88	80	42	26

Cell viability (%): 100, 75, 50, 25, 0

Quiescent-Senescent

IR-Q RPE-1		HHT (nM)					
		0	5	10	25	50	100
ABT-737 (µM)	0	100	103	98	98	96	93
	1	86	89	89	90	90	82
	2.5	78	78	83	86	81	58
	5	63	66	69	71	65	46
	10	50	48	51	55	44	34
	20	41	37	41	39	33	23

Synergy scoring (HSA) → **Synergy map**

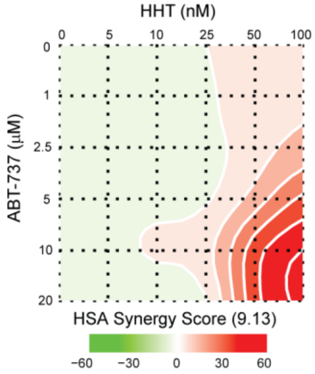

Difference between viability of proliferating and senescent cells → **Selective senolytic effect**

		HHT (nM)					
		0	5	10	25	50	100
ABT-737 (µM)	0						
	1					*	
	2.5		****	****	****	*	***
	5		****	****	****	***	***
	10		****	****	****	****	*
	20		****	****	****	***	

Synergy area for senescent cells

Selective senolytic area

Synergistic and selective senolytic effect

** Statistically significant reduced viability of senescent cells

** Statistically significant reduced viability of quiescent cells

Synergy scoring (HSA) → **Synergy map**

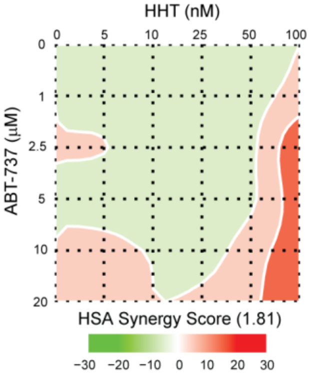

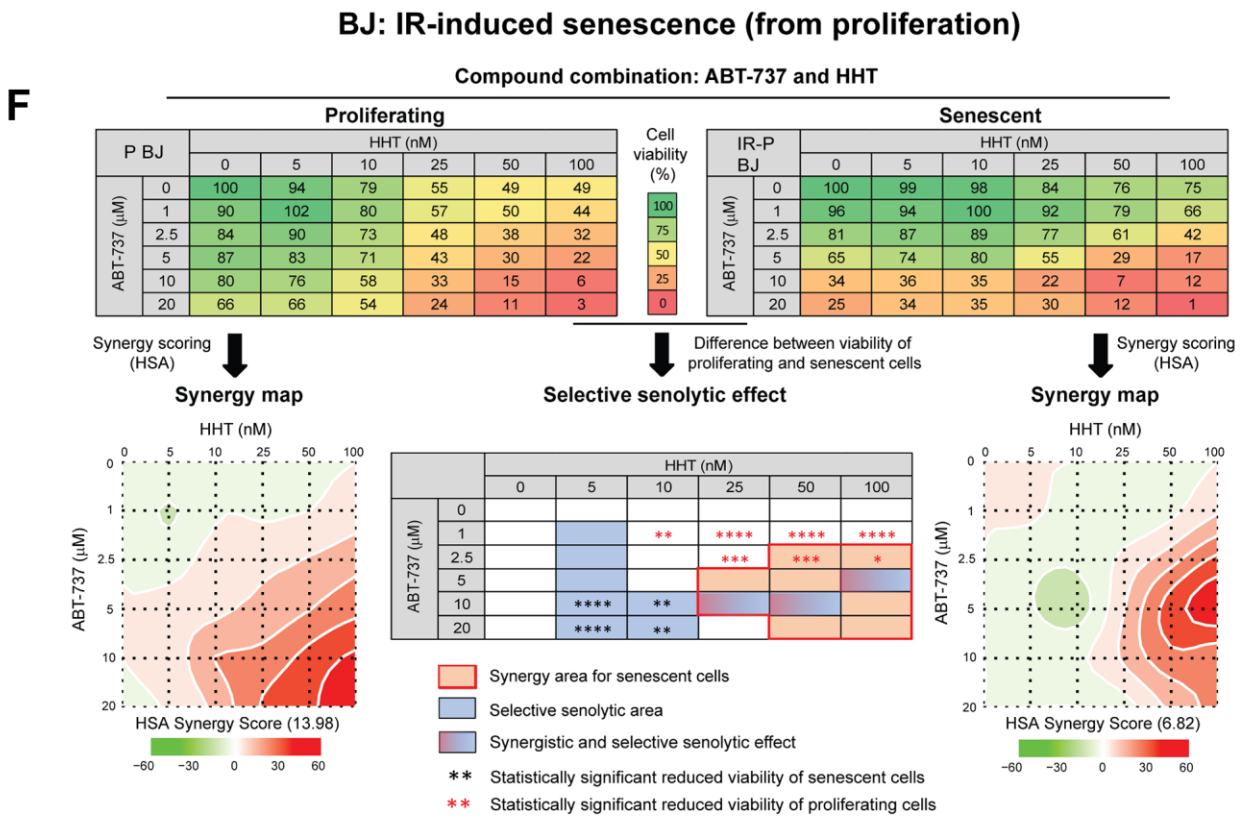
BJ: IR-induced senescence (from proliferation)
F
Compound combination: ABT-737 and HHT
Proliferating
P BJ
HHT (nM): 0 5 10 25 50 100
ABT-737 (μM)
0: 100 94 79 55 49 49
1: 90 102 80 57 50 44
2.5: 84 90 73 48 38 32
5: 87 83 71 43 30 22
10: 80 76 58 33 15 6
20: 66 66 54 24 11 3
Cell viability (%)
100 75 50 25 0
Senescent
IR-P BJ
HHT (nM): 0 5 10 25 50 100
ABT-737 (μM)
0: 100 99 98 84 76 75
1: 96 94 100 92 79 66
2.5: 81 87 89 77 61 42
5: 65 74 80 55 29 17
10: 34 36 35 22 7 12
20: 25 34 35 30 12 1
Synergy scoring (HSA)
Difference between viability of proliferating and senescent cells
Synergy map
Selective senolytic effect
HHT (nM)
ABT-737 (μM)
HSA Synergy Score (13.98)
HSA Synergy Score (6.82)
−60 −30 0 30 60
1: ** **** **** ****
2.5: *** *** *
10: **** **
20: **** **
Synergy area for senescent cells
Selective senolytic area
Synergistic and selective senolytic effect
** Statistically significant reduced viability of senescent cells
** Statistically significant reduced viability of proliferating cells

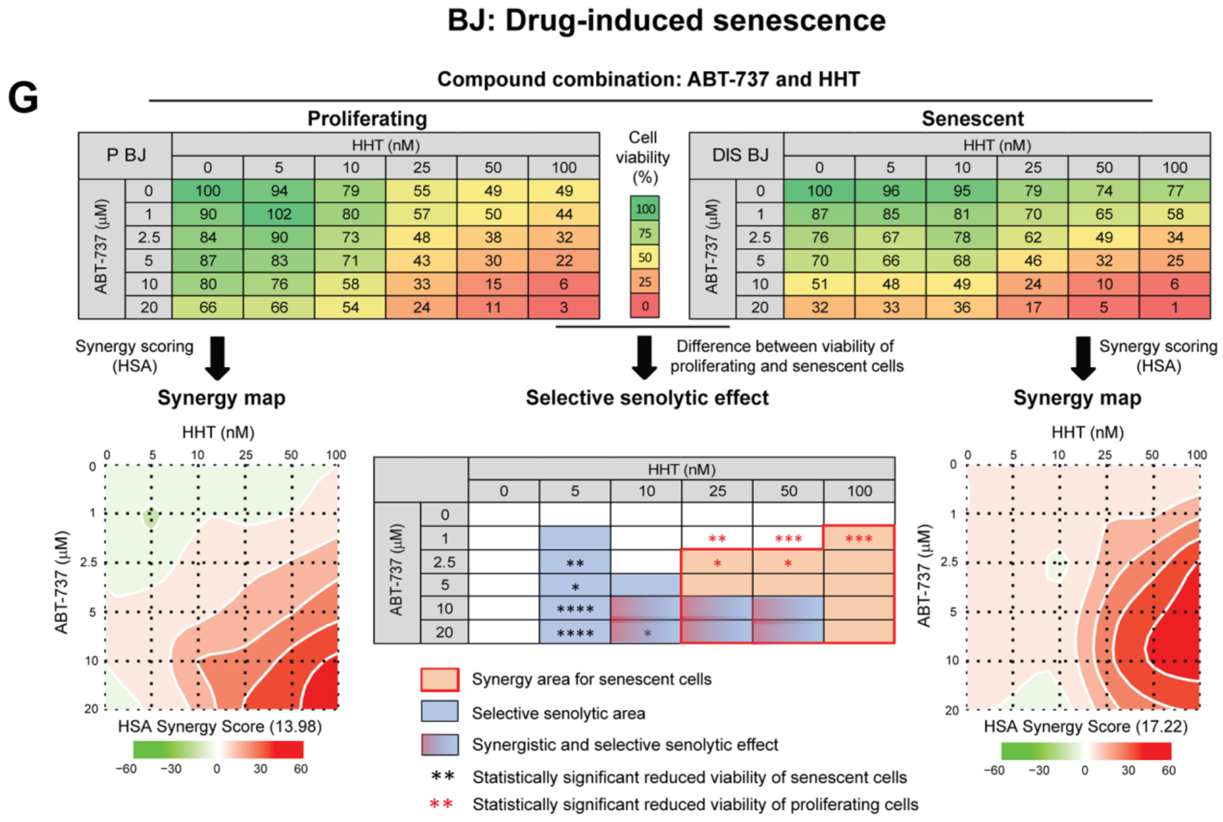
BJ: Drug-induced senescence
G
Compound combination: ABT-737 and HHT
Proliferating
P BJ
HHT (nM): 0 5 10 25 50 100
ABT-737 (μM)
0: 100 94 79 55 49 49
1: 90 102 80 57 50 44
2.5: 84 90 73 48 38 32
5: 87 83 71 43 30 22
10: 80 76 58 33 15 6
20: 66 66 54 24 11 3
Cell viability (%)
100 75 50 25 0
Senescent
DIS BJ
HHT (nM): 0 5 10 25 50 100
ABT-737 (μM)
0: 100 96 95 79 74 77
1: 87 85 81 70 65 58
2.5: 76 67 78 62 49 34
5: 70 66 68 46 32 25
10: 51 48 49 24 10 6
20: 32 33 36 17 5 1
Synergy scoring (HSA)
Difference between viability of proliferating and senescent cells
Synergy map
Selective senolytic effect
HHT (nM)
ABT-737 (μM)
HSA Synergy Score (13.98)
HSA Synergy Score (17.22)
−60 −30 0 30 60
1: ** *** ***
2.5: ** * *
5: *
10: ****
20: **** *
Synergy area for senescent cells
Selective senolytic area
Synergistic and selective senolytic effect
** Statistically significant reduced viability of senescent cells
** Statistically significant reduced viability of proliferating cells

H

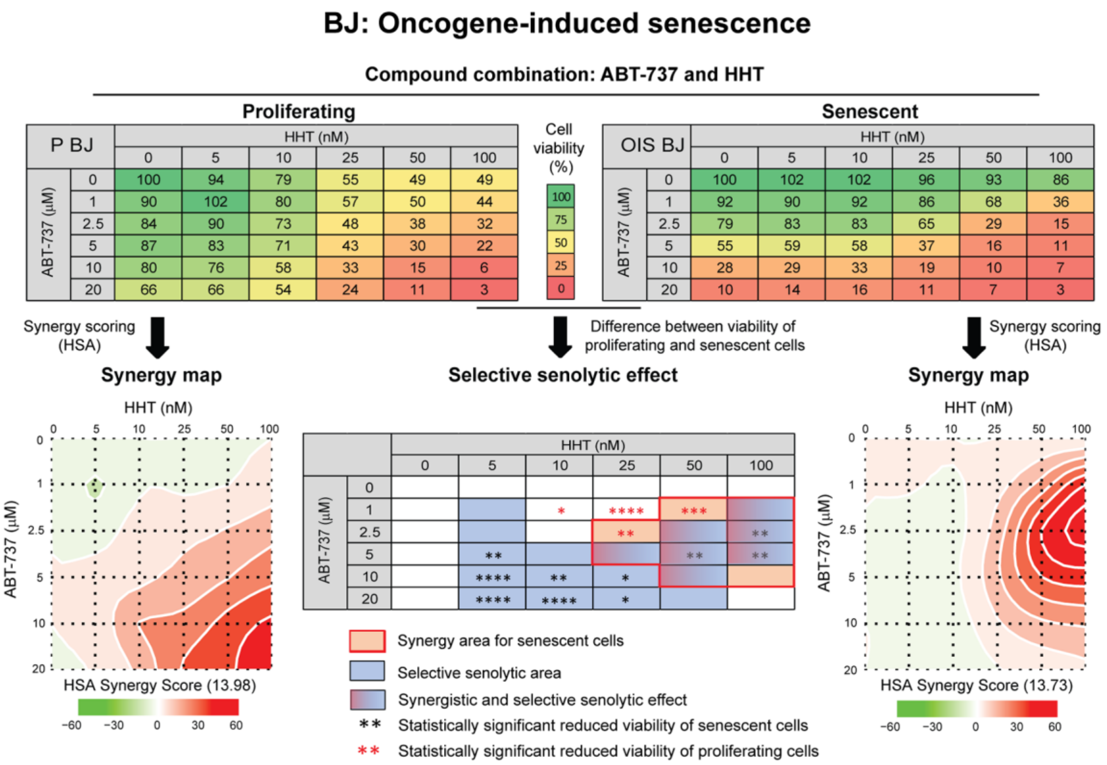

Proliferating (P BJ), cell viability (%)

ABT-737 (µM) \ HHT (nM)	0	5	10	25	50	100
0	100	94	79	55	49	49
1	90	102	80	57	50	44
2.5	84	90	73	48	38	32
5	87	83	71	43	30	22
10	80	76	58	33	15	6
20	66	66	54	24	11	3

Senescent (OIS BJ), cell viability (%)

ABT-737 (µM) \ HHT (nM)	0	5	10	25	50	100
0	100	102	102	96	93	86
1	92	90	92	86	68	36
2.5	79	83	83	65	29	15
5	55	59	58	37	16	11
10	28	29	33	19	10	7
20	10	14	16	11	7	3

Selective senolytic effect

ABT-737 (µM) \ HHT (nM)	0	5	10	25	50	100
0						
1			*	****	***	
2.5				**		**
5		**			**	**
10		****	**	*		
20		****	****	*		

I

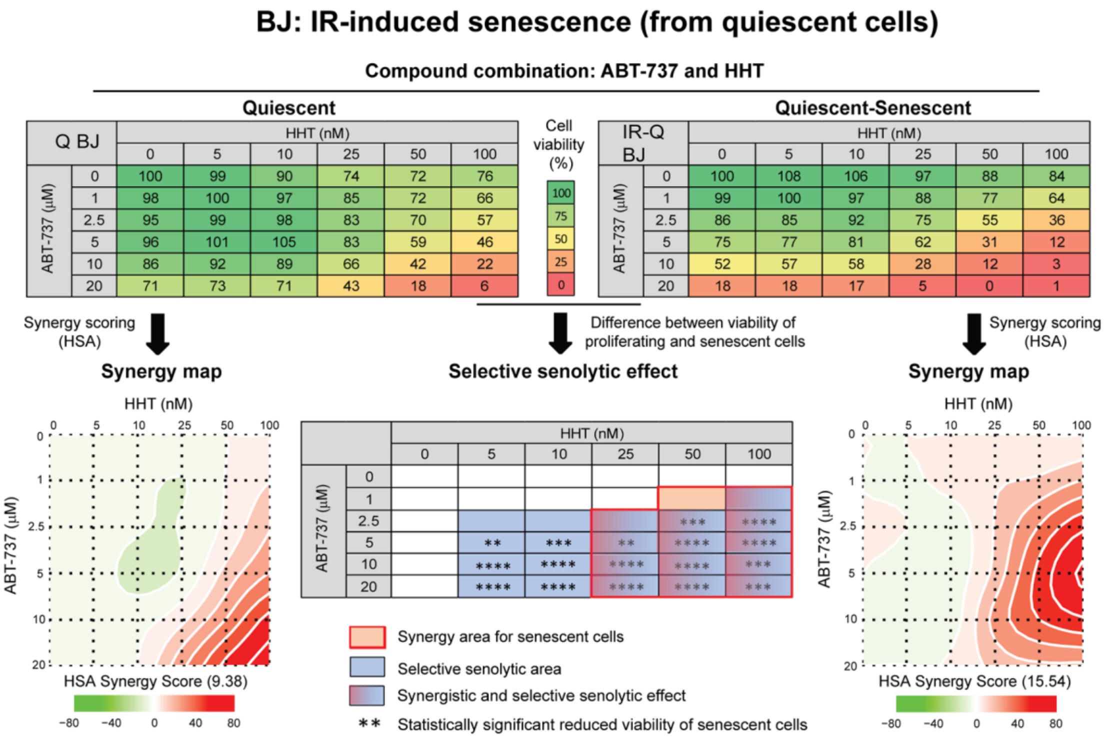

Quiescent (Q BJ), cell viability (%)

ABT-737 (µM) \ HHT (nM)	0	5	10	25	50	100
0	100	99	90	74	72	76
1	98	100	97	85	72	66
2.5	95	99	98	83	70	57
5	96	101	105	83	59	46
10	86	92	89	66	42	22
20	71	73	71	43	18	6

Quiescent-Senescent (IR-Q BJ), cell viability (%)

ABT-737 (µM) \ HHT (nM)	0	5	10	25	50	100
0	100	108	106	97	88	84
1	99	100	97	88	77	64
2.5	86	85	92	75	55	36
5	75	77	81	62	31	12
10	52	57	58	28	12	3
20	18	18	17	5	0	1

Selective senolytic effect

ABT-737 (µM) \ HHT (nM)	0	5	10	25	50	100
0						
1						
2.5					***	****
5		**	***	**	****	****
10		****	****	****	****	***
20		****	****	****	****	***

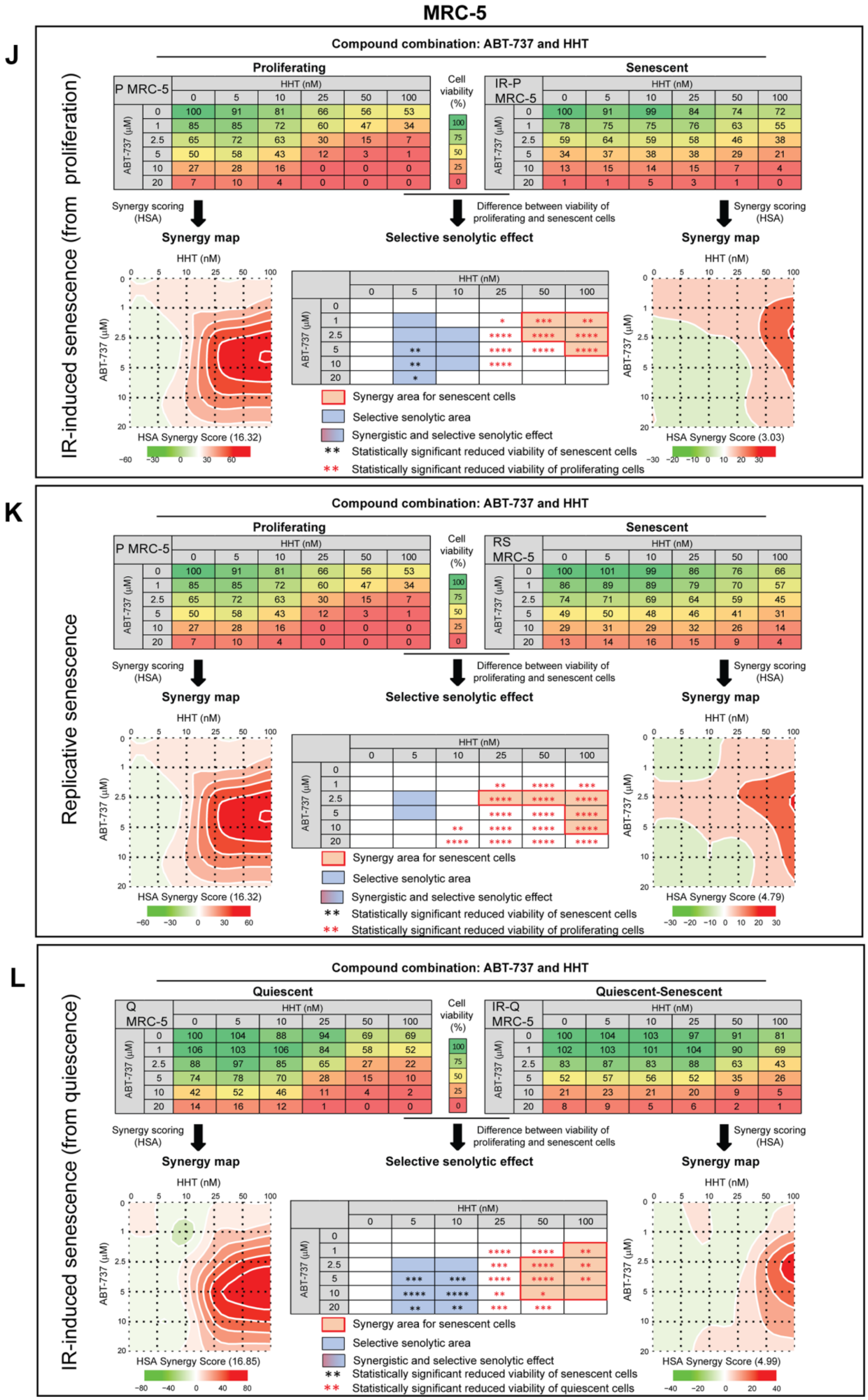
MRC-5
J
IR-induced senescence (from proliferation)
Compound combination: ABT-737 and HHT
Proliferating
P MRC-5
HHT (nM): 0 5 10 25 50 100
ABT-737 (μM)
0: 100 91 81 66 56 53
1: 85 85 72 60 47 34
2.5: 65 72 63 30 15 7
5: 50 58 43 12 3 1
10: 27 28 16 0 0 0
20: 7 10 4 0 0 0
Cell viability (%)
100 75 50 25 0
Senescent
IR-P MRC-5
HHT (nM): 0 5 10 25 50 100
0: 100 91 99 84 74 72
1: 78 75 75 76 63 55
2.5: 59 64 59 58 46 38
5: 34 37 38 38 29 21
10: 13 15 14 15 7 4
20: 1 1 5 3 1 0
Synergy scoring (HSA)
Difference between viability of proliferating and senescent cells
Synergy map
Selective senolytic effect
HSA Synergy Score (16.32)
HSA Synergy Score (3.03)
Synergy area for senescent cells
Selective senolytic area
Synergistic and selective senolytic effect
** Statistically significant reduced viability of senescent cells
** Statistically significant reduced viability of proliferating cells
K
Replicative senescence
Compound combination: ABT-737 and HHT
Proliferating
P MRC-5
HHT (nM): 0 5 10 25 50 100
0: 100 91 81 66 56 53
1: 85 85 72 60 47 34
2.5: 65 72 63 30 15 7
5: 50 58 43 12 3 1
10: 27 28 16 0 0 0
20: 7 10 4 0 0 0
Senescent
RS MRC-5
HHT (nM): 0 5 10 25 50 100
0: 100 101 99 86 76 66
1: 86 89 89 79 70 57
2.5: 74 71 69 64 59 45
5: 49 50 48 46 41 31
10: 29 31 29 32 26 14
20: 13 14 16 15 9 4
HSA Synergy Score (16.32)
HSA Synergy Score (4.79)
L
IR-induced senescence (from quiescence)
Compound combination: ABT-737 and HHT
Quiescent
Q MRC-5
HHT (nM): 0 5 10 25 50 100
0: 100 104 88 94 69 69
1: 106 103 106 84 58 52
2.5: 88 97 85 65 27 22
5: 74 78 70 28 15 10
10: 42 52 46 11 4 2
20: 14 16 12 1 0 0
Quiescent-Senescent
IR-Q MRC-5
HHT (nM): 0 5 10 25 50 100
0: 100 104 103 97 91 81
1: 102 103 101 104 90 69
2.5: 83 87 83 88 63 43
5: 52 57 56 52 35 26
10: 21 23 21 20 9 5
20: 8 9 5 6 2 1
HSA Synergy Score (16.85)
HSA Synergy Score (4.99)
** Statistically significant reduced viability of quiescent cells

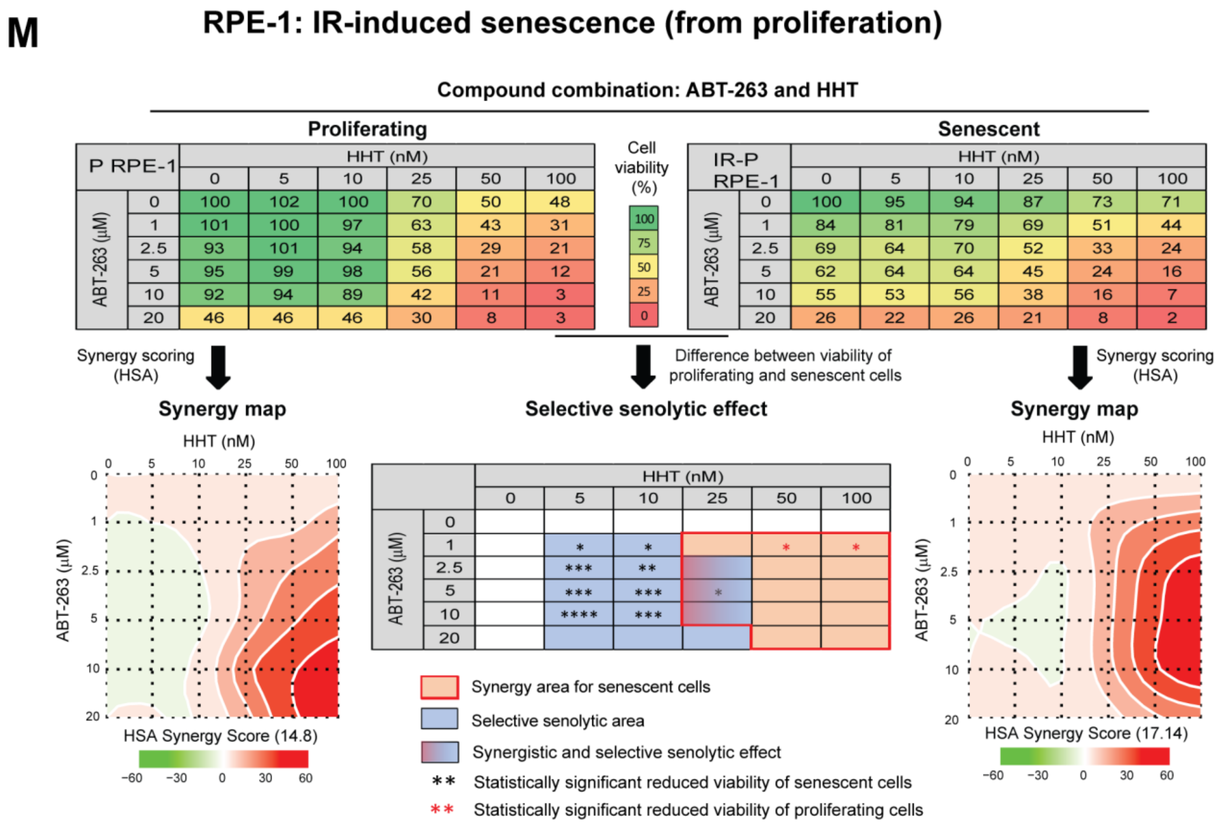

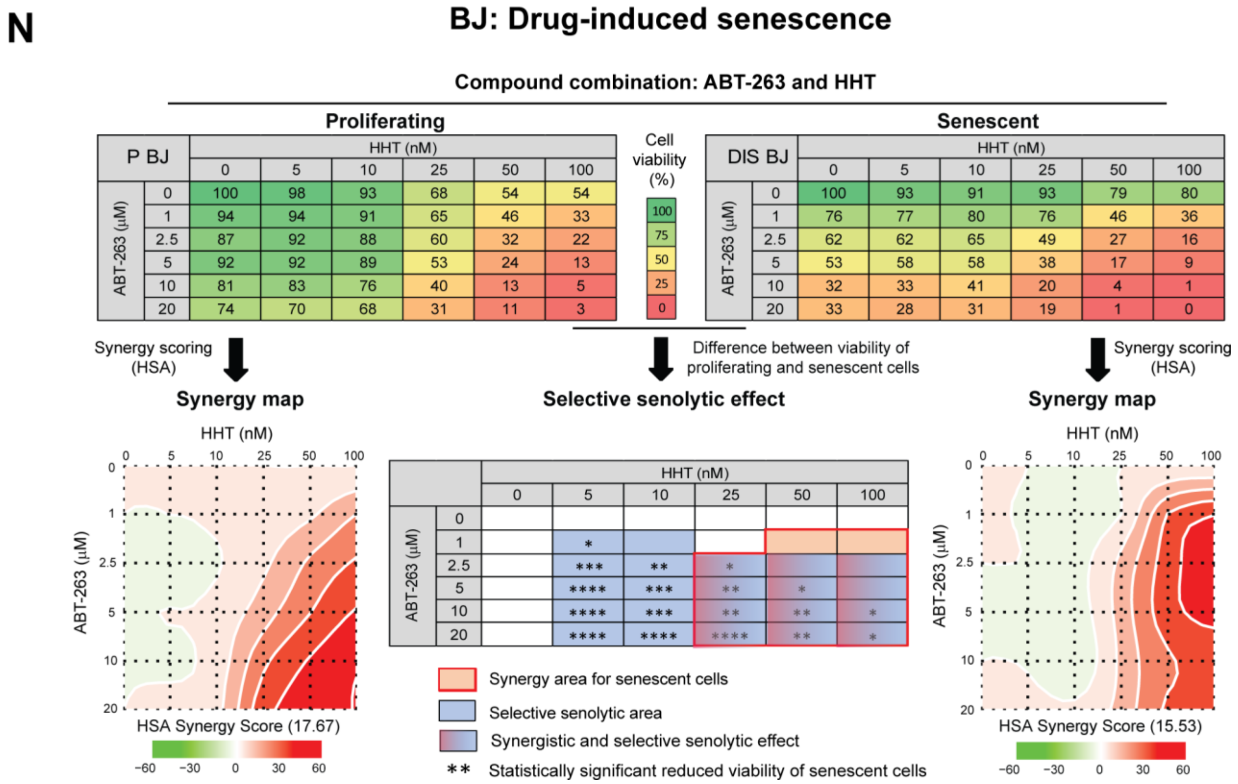

Supplementary Figure 2. The effect of homoharringtonine, ABT-737, ABT-263, and their combinations on the viability of senescent, quiescent, and proliferating cells. Senescent cells and their appropriate controls were treated for 24 hours either with a

single compound (**A**–**C**) or with their combinations (**D**–**N**). The residual viability was assessed by staining with crystal violet and presented as a percentage of untreated control. For treatment with HHT (**A**), ABT-737 (**B**), and ABT-263 (**C**), the viability was expressed as the mean ± S.D. from at least three independent experiments and plotted in histograms. The statistical analysis in panels A – C was carried out using two way ANOVA; ns. = no significant difference, P > 0.05; **, P < 0.01; ***, P < 0.001. The results for treatment with a combination of two compounds are expressed as matrices of viabilities with color scales inside (dark green, 100% viability; orange, 50% viability; dark red, 0% viability) and the diagram of the synergy score (SC; SC < -10: the interaction between two drugs is likely to be antagonistic; -10 < SC< 10: the interaction between two drugs is likely to be additive; SC>10: the interaction between two drugs is likely to be synergistic) both for control and senescent models. Next, the matrix showing the region with synergistic and selective senolytic effect and its statistical significance (two-tailed Student's t-test; *, P > 0.05; **, P < 0.01; ***, P < 0.001) is shown. This scheme presents the cytotoxic and senolytic effect of ABT-737 and HHT on IR-induced senescence from proliferation (IR-P) in RPE-1 (**D**), IR-induced senescence from quiescence (IR-Q) in RPE-1 (**E**), IR-P BJ (**F**), drug-induced senescence (DIS) in BJ (**G**), oncogene-induce senescence (OIS) in BJ (**H**), IR-Q BJ (**I**), IR-P MRC-5 (**J**), replicative senescence (RS) in MRC-5 (**K**), and IR-Q MRC-5 (**L**). The same scheme was also used to present the senolytic effect of ABT-263 and HHT on IR-P RPE-1 (**M**) and DIS BJ (**N**) cells.

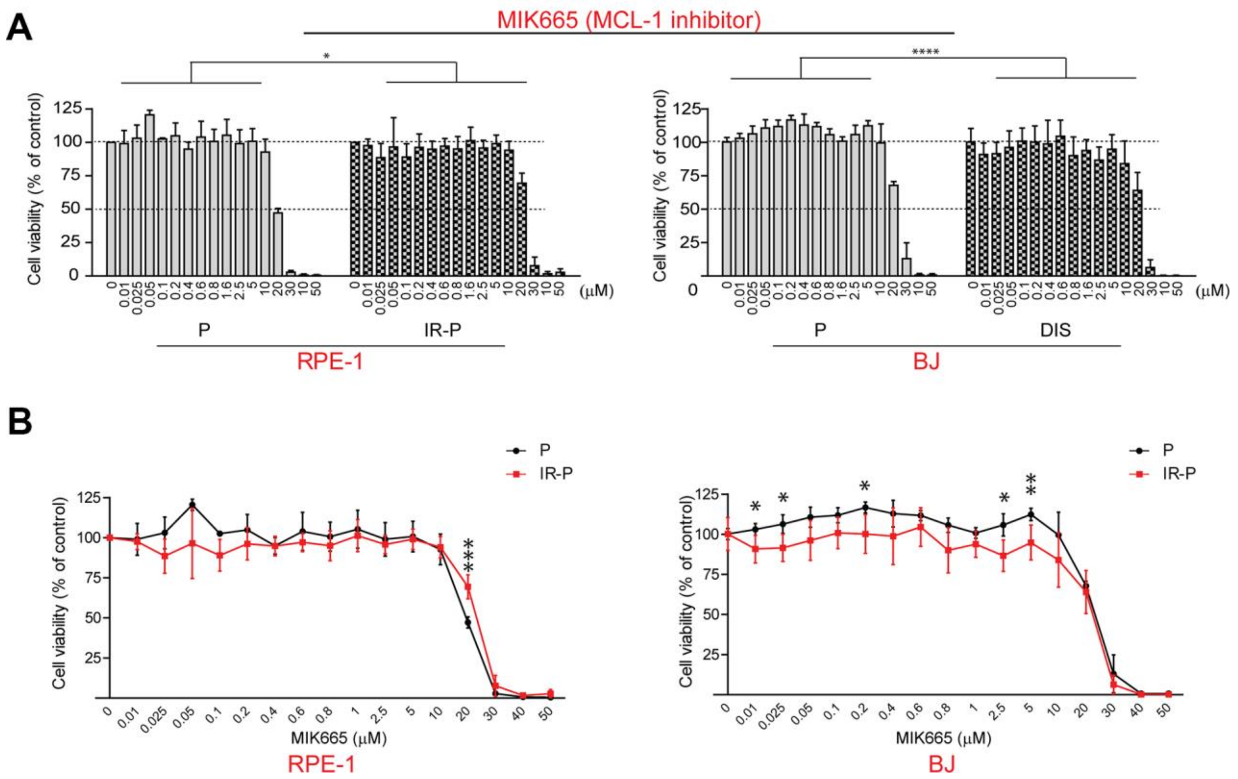
A
MIK665 (MCL-1 inhibitor)
Cell viability (% of control)
P
IR-P
RPE-1
DIS
BJ
(μM)
B
MIK665 (μM)
RPE-1
BJ

C

RPE: IR-induced senescence (from proliferation)

Compound combination: ABT-737 and MIK665

Proliferating

P RPE-1 — ABT-737 (μM) \ MIK665 (μM)	0	0.01	0.025	0.05	0.1	0.2	0.4	0.6	0.8	1	2.5	5	10	20	30
0	100	99	103	105	103	101	95	98	99	106	99	96	92	49	3
0.2	98	103	110	104	106	100	99	98	97	98	60	46	31	0	0
0.4	99	107	101	105	100	95	89	79	71	65	19	7	4	3	6
0.6	93	105	105	107	103	91	78	64	48	40	4	3	2	2	4
0.8	98	106	106	105	107	80	59	41	26	20	1	2	1	2	2
1	99	108	102	99	98	72	48	34	20	13	5	2	2	2	4
2.5	98	103	70	64	39	14	3	1	0	1	2	1	2	1	3
5	97	54	12	6	1	0	1	1	0	1	1	1	3	1	3
10	85	3	0	1	0	0	0	0	0	0	0	1	1	0	1
20	70	0	0	0	0	0	0	0	0	0	0	1	0	0	0

Cell viability (%): 100, 75, 50, 25, 0

Senescent

IR-P RPE-1 — ABT-737 (μM) \ MIK665 (μM)	0	0.01	0.025	0.05	0.1	0.2	0.4	0.6	0.8	1	2.5	5	10	20	30
0	100	97	88	96	89	97	95	97	95	104	96	99	91	63	6
0.2	93	104	105	112	104	93	92	91	86	83	46	45	43	0	0
0.4	90	103	93	101	102	88	77	67	61	55	23	19	3	0	3
0.6	92	98	104	107	99	74	51	42	31	24	5	5	6	5	4
0.8	94	95	87	110	84	57	34	19	14	9	0	0	0	0	0
1	87	92	76	92	65	46	20	12	7	5	5	6	3	3	5
2.5	77	57	35	29	12	5	3	1	2	1	5	5	2	4	4
5	60	14	5	5	1	1	2	1	2	2	5	4	2	2	3
10	30	3	1	3	0	0	0	0	1	0	2	2	1	0	0
20	9	0	0	1	0	0	0	0	0	0	0	0	0	0	0

Synergy scoring (HSA) | Difference between viability of proliferating and senescent cells | Synergy scoring (HSA)

Synergy map

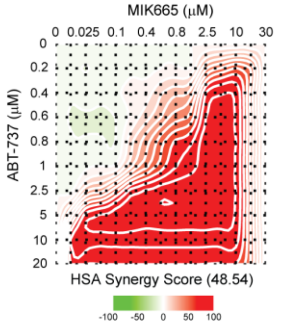

Selective senolytic effect

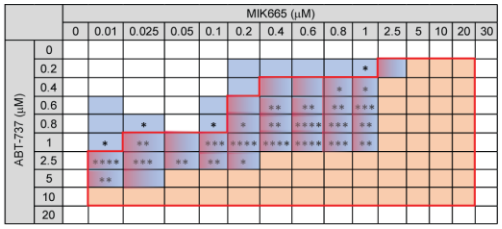

Synergy map

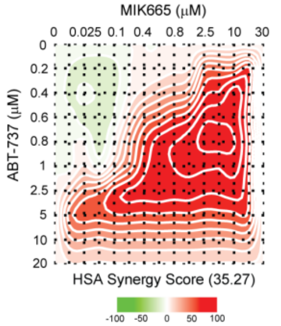

Synergy area for senescent cells

Selective senolytic area

Synergistic and selective senolytic effect

** Statistically significant reduced viability of senescent cells

D

BJ: Drug-induced senescence

Compound combination: ABT-737 and MIK665

Proliferating

P BJ — ABT-737 (μM) \ MIK665 (μM)	0	0.01	0.025	0.05	0.1	0.2	0.4	0.6	0.8	1	2.5	5	10	20	30
0	100	103	106	112	114	125	116	118	106	101	108	114	101	68	13
0.2	107	108	107	104	107	105	102	103	106	96	72	55	41	4	1
0.4	114	109	105	104	105	105	96	86	73	58	20	9	3	4	5
0.6	103	105	109	107	106	103	75	57	42	29	3	2	1	4	4
0.8	104	107	110	108	106	89	60	41	22	8	0	1	1	4	2
1	107	102	106	105	98	71	36	25	13	2	0	0	0	3	2
2.5	95	75	50	41	26	11	9	2	2	2	2	3	3	3	5
5	94	31	9	6	3	2	2	2	1	2	2	3	4	3	5
10	87	3	4	2	1	0	0	1	4	0	0	1	3	2	4
20	44	0	1	0	1	0	0	1	1	1	0	0	1	1	2

Cell viability (%): 100, 75, 50, 25, 0

Senescent

DIS BJ — ABT-737 (μM) \ MIK665 (μM)	0	0.01	0.025	0.05	0.1	0.2	0.4	0.6	0.8	1	2.5	5	10	20	30
0	100	91	92	98	101	107	102	110	90	94	87	95	84	64	6
0.2	83	100	95	95	102	96	85	84	79	79	47	50	41	6	0
0.4	83	102	84	82	82	80	71	65	50	48	24	21	1	0	2
0.6	91	86	84	80	78	59	53	42	36	21	8	8	2	2	1
0.8	76	80	72	69	58	46	37	24	12	12	0	0	0	0	2
1	70	68	58	56	39	34	13	12	3	1	0	0	0	0	0
2.5	54	51	28	33	17	9	4	1	4	3	4	3	1	1	1
5	30	9	3	6	4	1	3	1	2	2	4	2	1	1	2
10	24	3	2	2	0	0	0	0	1	0	1	1	0	0	0
20	13	0	0	0	0	0	0	0	0	0	0	0	0	0	0

Synergy scoring (HSA) | Difference between viability of proliferating and senescent cells | Synergy scoring (HSA)

Synergy map

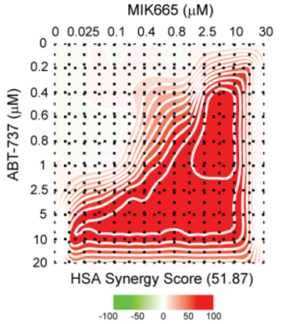

Selective senolytic effect

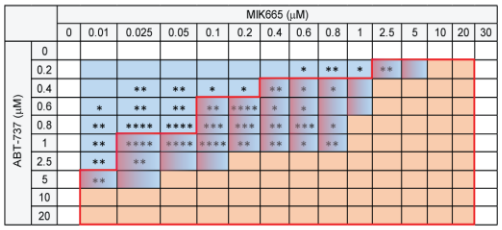

Synergy map

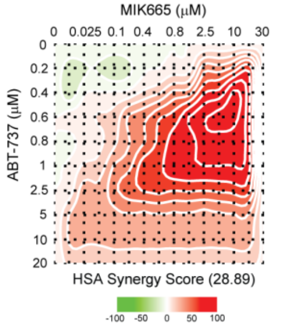

Synergy area for senescent cells

Selective senolytic area

Synergistic and selective senolytic effect

** Statistically significant reduced viability of senescent cells

E

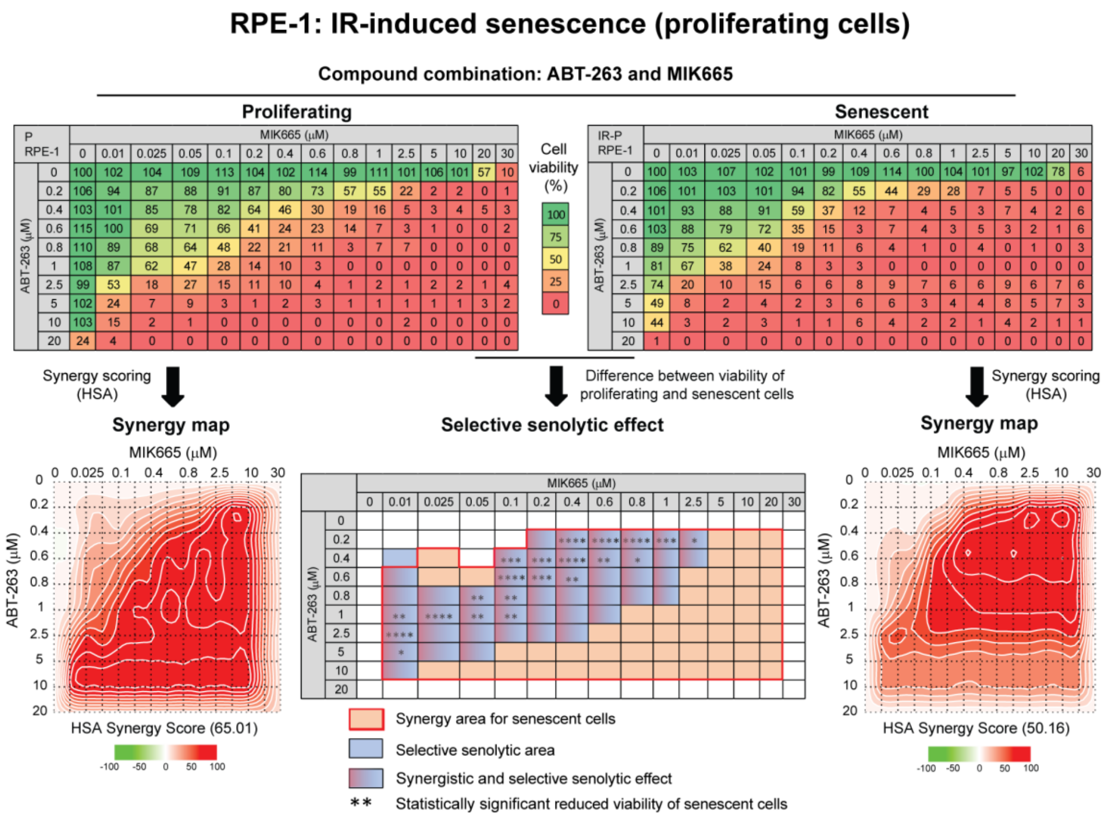

F

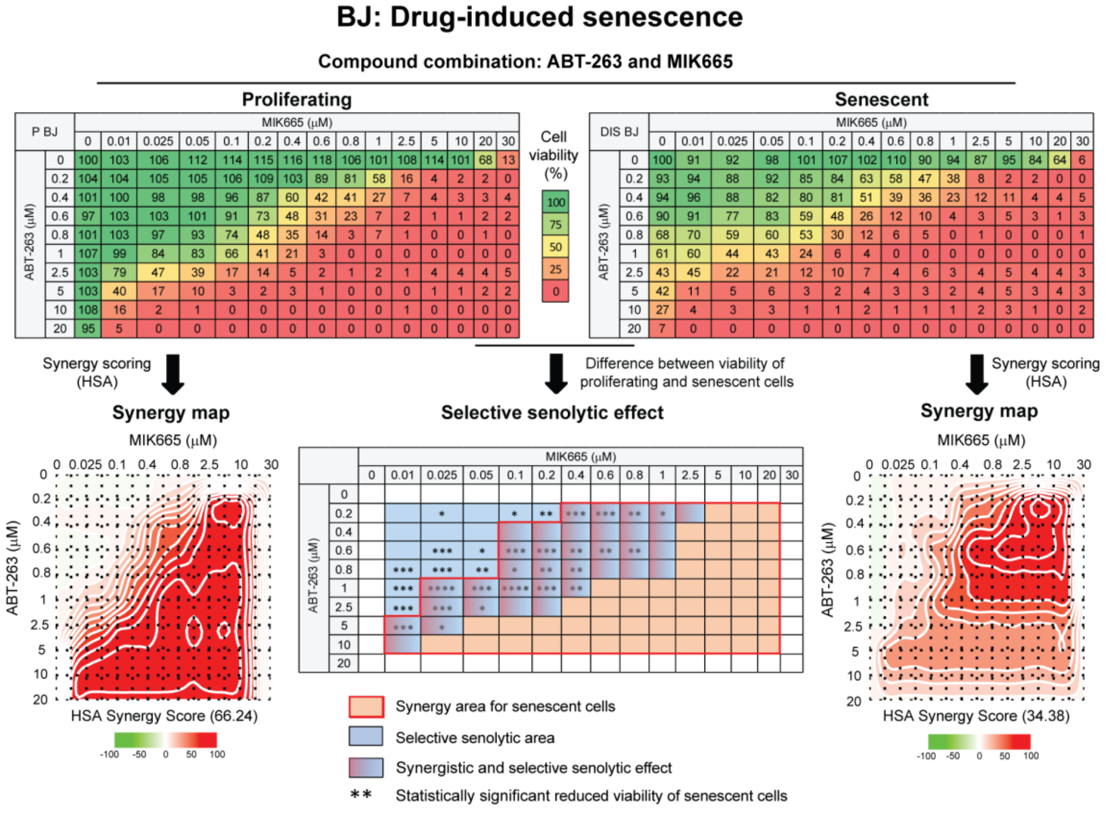

Supplementary Figure 3. The effect of MIK665 and its combinations with ABT-737 and ABT-263 on the viability of senescent and proliferating cells. Senescent cells and their appropriate controls were treated for 24 hours either with MIK665 (**A**, **B**) or in

combination with ABT-737 (**C**, **D**) or ABT-263 (**E**, **F**). The residual viability was assessed by staining with crystal violet and presented as a percentage of untreated control. For treatment with MIK665 alone, the viability was expressed as the mean ± S.D. from at least three independent experiments and plotted in histograms (**A**) or line graph (**B**). The statistical analysis in panel A was carried out using two way ANOVA, in panel B using t-test; P > 0.05; **, P < 0.01; ***, P < 0.001; ****, P < 0.0001. The results for treatment with a combination of two compounds are expressed as matrices of viabilities with color scales inside (dark green, 100% viability; orange, 50% viability; dark red, 0% viability) and the diagram of the synergy score (SC; SC < -10: the interaction between two drugs is likely to be antagonistic; -10 < SC< 10: the interaction between two drugs is likely to be additive; SC>10: the interaction between two drugs is likely to be synergistic) both for control and senescent models. Next, the matrix showing the region with synergistic and selective senolytic effect and its statistical significance (two-tailed Student's t-test; *, P > 0.05; **, P < 0.01; ***, P < 0.001; ****, P < 0.0001) is shown. This scheme presents the cytotoxic and senolytic effect of ABT-737 and MIK665 on IR-induced senescence from proliferation (IR-P) in RPE-1 (**C**), and drug-induced senescence (DIS) in BJ (**D**). The same scheme was also used for the presentation of the senolytic effect of ABT-263 and MIK665 on IR-P RPE-1 (**E**) and DIS BJ (**F**) cells.

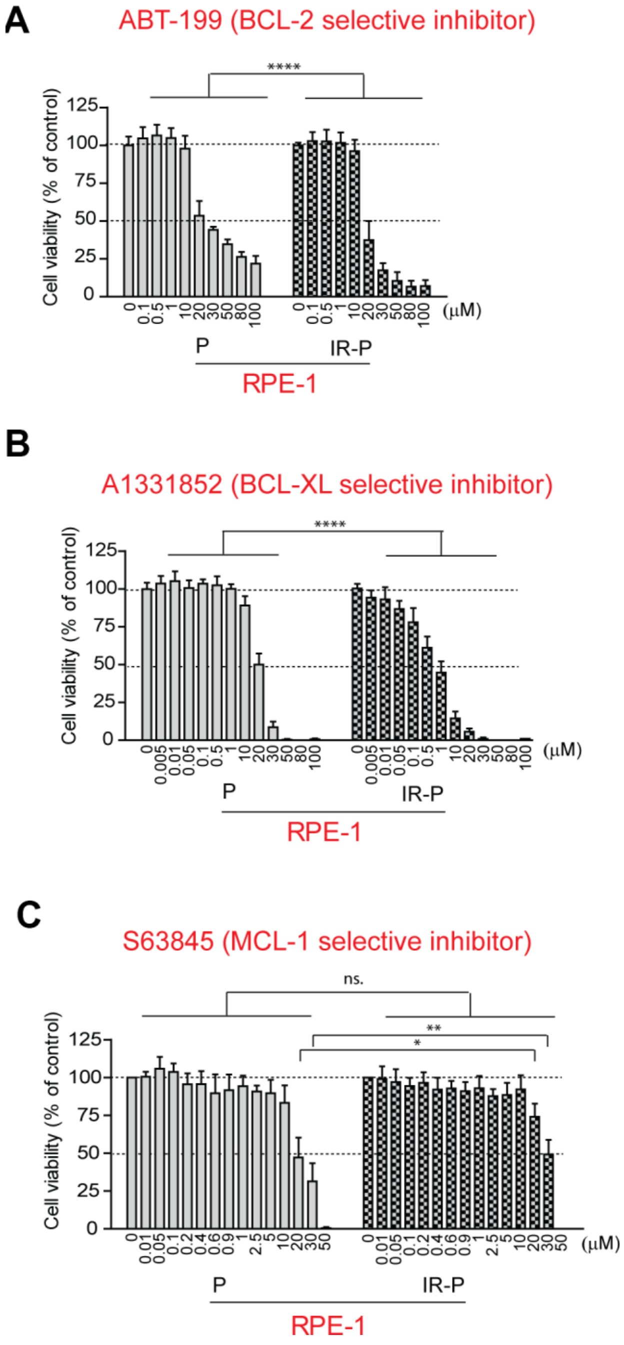
A
ABT-199 (BCL-2 selective inhibitor)
****
Cell viability (% of control)
125
100
75
50
25
0
0 0.1 0.5 1 10 20 30 50 80 100
0 0.1 0.5 1 10 20 30 50 80 100 (μM)
P
IR-P
RPE-1
B
A1331852 (BCL-XL selective inhibitor)
****
Cell viability (% of control)
125
100
75
50
25
0
0 0.005 0.01 0.05 0.1 0.5 1 10 20 30 50 80 100
0 0.005 0.01 0.05 0.1 0.5 1 10 20 30 50 80 100 (μM)
P
IR-P
RPE-1
C
S63845 (MCL-1 selective inhibitor)
ns.
**
*
Cell viability (% of control)
125
100
75
50
25
0
0 0.01 0.05 0.1 0.2 0.4 0.6 0.9 1 2.5 5 10 20 30 50
0 0.01 0.05 0.1 0.2 0.4 0.6 0.9 1 2.5 5 10 20 30 50 (μM)
P
IR-P
RPE-1

D

Compound combination: ABT-199 and S63845

Proliferating

P RPE-1 ABT-199 (μM) \ S63845 (μM)	0	0.2	0.4	0.6	0.8	1	2.5	5	10	20	30
0	100	96	96	89	92	94	91	90	83	47	32
0.5	91	89	88	89	86	93	83	86	86	52	16
1	101	98	92	92	95	93	85	86	84	54	7
2.5	90	87	83	92	89	99	91	76	74	30	3
5	97	89	85	87	82	80	53	43	18	8	5
10	83	76	68	61	54	48	17	9	4	3	2
20	72	28	18	14	11	9	4	2	2	3	3
30	42	8	4	3	3	3	2	2	1	2	2

Cell viability (%): 100, 75, 50, 25, 0

Senescent

IR-P RPE-1 ABT-199 (μM) \ S63845 (μM)	0	0.2	0.4	0.6	0.8	1	2.5	5	10	20	30
0	100	96	92	93	91	93	88	88	92	74	49
0.5	95	91	92	90	86	88	85	89	89	67	43
1	101	92	93	94	90	84	91	95	102	79	33
2.5	95	85	87	90	92	93	89	86	78	45	21
5	97	84	80	76	74	70	49	37	27	5	7
10	91	56	41	34	28	25	10	4	3	1	4
20	70	6	3	3	2	3	1	1	1	0	3
30	14	1	1	1	0	1	0	1	1	1	1

Synergy scoring (HSA) → **Synergy map**

Difference between viability of proliferating and senescent cells → **Selective senolytic effect**

Synergy scoring (HSA) → **Synergy map**

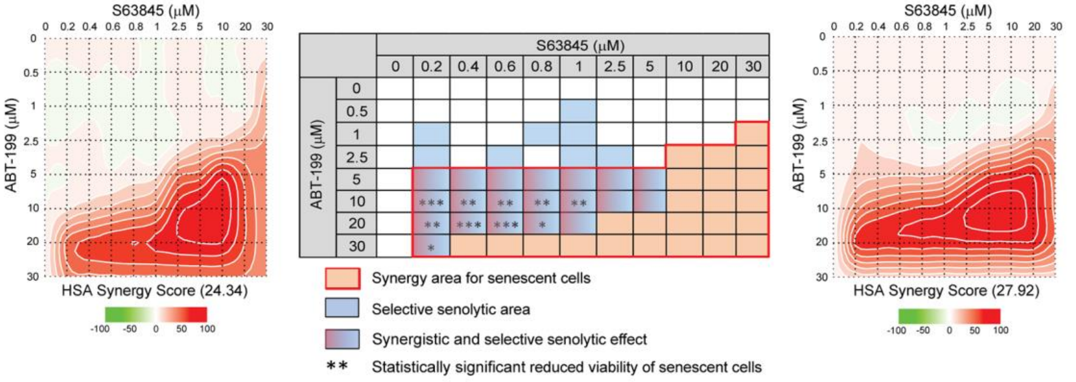

E

Compound combination: A1331852 and S63845

Proliferating

P RPE-1 A1331852 (μM) \ S63845 (μM)	0	0.01	0.05	0.1	0.2	0.4	0.6	0.8	1	2.5	5	10	20	30
0	100	100	106	103	96	96	89	92	94	91	90	83	47	32
0.001	95	103	101	96	100	102	105	106	97	91	70	35	7	5
0.005	97	107	108	101	102	101	104	97	96	82	29	7	1	1
0.01	100	103	115	105	105	98	99	95	87	58	8	2	1	2
0.05	97	96	98	100	90	70	49	35	27	7	1	0	0	0
0.1	100	101	102	98	71	47	32	17	12	2	1	0	0	0
0.5	95	92	73	52	21	10	5	2	2	1	1	0	0	0
1	97	84	37	23	10	3	1	1	1	1	0	1	0	0
2.5	87	51	9	5	1	0	0	0	0	0	0	0	0	0
5	89	29	7	1	1	0	0	0	1	1	1	0	0	0
10	81	22	3	1	0	0	0	0	0	0	0	0	1	0

Cell viability (%): 100, 75, 50, 25, 0

Senescent

IR-P RPE-1 A1331852 (μM) \ S63845 (μM)	0	0.01	0.05	0.1	0.2	0.4	0.6	0.8	1	2.5	5	10	20	30
0	100	99	97	94	96	92	93	91	93	88	88	92	74	49
0.001	95	88	90	88	87	87	90	86	88	89	87	84	51	9
0.005	88	91	86	89	84	83	80	75	80	77	64	44	15	2
0.01	82	85	85	83	77	82	75	69	69	52	30	13	3	2
0.05	78	81	80	80	56	37	25	16	13	4	2	0	0	0
0.1	74	83	73	65	37	19	11	7	5	2	1	0	4	0
0.5	58	55	19	8	2	2	1	1	1	1	1	0	0	0
1	46	26	4	2	1	1	1	0	1	1	0	0	1	1
2.5	32	9	1	0	1	0	0	0	0	0	0	0	2	0
5	22	5	2	2	1	0	0	0	0	0	0	3	0	0
10	17	2	1	2	1	0	0	0	0	0	1	0	0	0

Synergy scoring (HSA) → **Synergy map**

Difference between viability of proliferating and senescent cells → **Selective senolytic effect**

Synergy scoring (HSA) → **Synergy map**

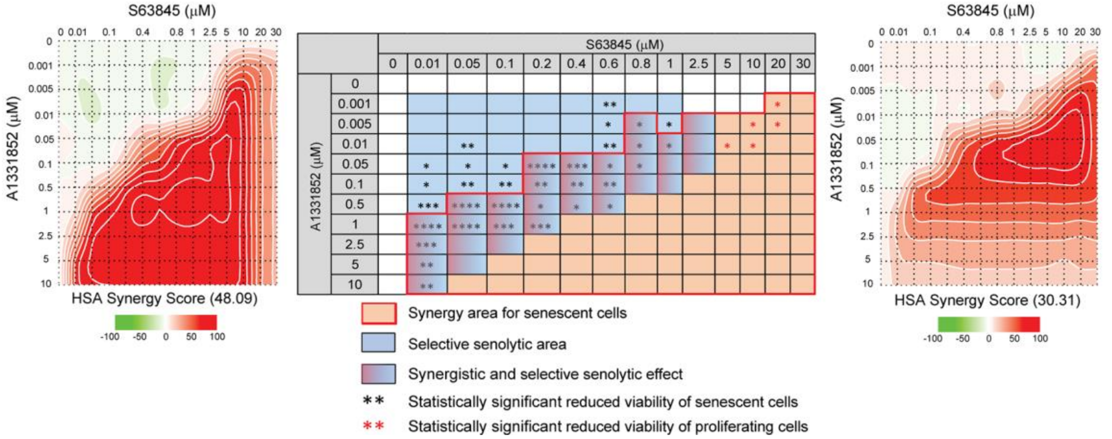

Supplementary Figure 4. The effect of ABT-199, A1331852, S63845, and their combinations on the viability of senescent, quiescent, and proliferating cells. Senescent cells and their appropriate controls were treated for 24 hours either with a single compound

(**A**–**C**) or with their combinations (**D**, **E**). The residual viability was assessed by staining with crystal violet and presented as a percentage of untreated control. The residual viability was assessed by staining with crystal violet and presented as a percentage of untreated control. For treatment with ABT199 (**A**), A1331852 (**B**), and S63845 (**C**), the viability was expressed as the mean ± S.D. from at least three independent experiments and plotted in histograms. The statistical analysis in panels A – C was carried out using two way ANOVA and two-tailed Student's t-test (**C**); ns. = no significant difference, P > 0.05; **, P < 0.01; ***, P < 0.001; ****, P < 0.0001. The results for treatment with a combination of two compounds are expressed as matrices of viabilities with color scales inside (dark green, 100% viability; orange, 50% viability; dark red, 0% viability) and the diagram of the synergy score (SC; SC < -10: the interaction between two drugs is likely to be antagonistic; -10 < SC< 10: the interaction between two drugs is likely to be additive; SC>10: the interaction between two drugs is likely to be synergistic) both for control and senescent models. Next, the matrix showing the region with synergistic and selective senolytic effect and its statistical significance (two-tailed Student's t-test; *, P > 0.05; **, P < 0.01; ***, P < 0.001; ****, P < 0.0001) is shown. This scheme presents the cytotoxic and senolytic effect of ABT-199 and S63845 (**C**) or A1331852 and S63845 (**D**) on IR-induced senescence from proliferation (IR-P) in RPE-1 cells.
